# Serum Metabolomic and Lipoprotein Profiling of Pancreatic Ductal Adenocarcinoma Patients of African Ancestry

**DOI:** 10.3390/metabo11100663

**Published:** 2021-09-28

**Authors:** Nnenna Elebo, Jones Omoshoro-Jones, Pascaline N. Fru, John Devar, Christiaan De Wet van Zyl, Barend Christiaan Vorster, Martin Smith, Stefano Cacciatore, Luiz F. Zerbini, Geoffrey Candy, Ekene Emmanuel Nweke

**Affiliations:** 1 Department of Surgery, School of Clinical Medicine, Faculty of Health Sciences, University of the Witwatersrand, Johannesburg 2193, South Africa; 500688@students.wits.ac.za (N.E.); omsjon@dr.com (J.O.-J.); pascaline.fru@wits.ac.za (P.N.F.); devarjohn@yahoo.com (J.D.); martin.smith@wits.ac.za (M.S.); geoffrey.candy@wits.ac.za (G.C.); 2 Hepatopancreatobiliary Unit, Department of Surgery, Chris Hani-Baragwanath Academic Hospital, Johannesburg 1864, South Africa; 3 Centre for Human Metabolomics, Faculty of Natural and Agricultural Sciences, North-West University, Potchefstroom 2531, South Africa; 22130438@nwu.ac.za (C.D.W.v.Z.); chris.vorster@nwu.ac.za (B.C.V.); 4 Cancer Genomics Group, International Centre for Genetic Engineering and Biotechnology, Cape Town 7925, South Africa; Stefano.Cacciatore@icgeb.org (S.C.); luiz.zerbini@icgeb.org (L.F.Z.); 5 Institute for Reproductive and Developmental Biology, Imperial College, London SW7 2AZ, UK

**Keywords:** pancreatic ductal adenocarcinoma, metabolites, cholestatic (obstructive) jaundice, lipoprotein, inflammation, tumour stages

## Abstract

Pancreatic ductal adenocarcinoma (PDAC) is a lethal cancer with a characteristic dysregulated metabolism. Abnormal clinicopathological features linked to defective metabolic and inflammatory response pathways can induce PDAC development and progression. In this study, we investigated the metabolites and lipoproteins profiles of PDAC patients of African ancestry. Nuclear Magnetic Resonance (NMR) spectroscopy was conducted on serum obtained from consenting individuals (34 PDAC, 6 Chronic Pancreatitis, and 6 healthy participants). Seventy-five signals were quantified from each NMR spectrum. The Liposcale test was used for lipoprotein characterization. Spearman’s correlation and Kapan Meier tests were conducted for correlation and survival analyses, respectively. In our patient cohort, the results demonstrated that levels of metabolites involved in the glycolytic pathway increased with the tumour stage. Raised ethanol and 3-hydroxybutyrate were independently correlated with a shorter patient survival time, irrespective of tumour stage. Furthermore, increased levels of bilirubin resulted in an abnormal lipoprotein profile in PDAC patients. Additionally, we observed that the levels of a panel of metabolites (such as glucose and lactate) and lipoproteins correlated with those of inflammatory markers. Taken together, the metabolic phenotype can help distinguish PDAC severity and be used to predict patient survival and inform treatment intervention.

## 1. Introduction

Pancreatic Ductal Adenocarcinoma (PDAC) is one of the most fatal cancers, primarily due to its late-stage presentation and resistance to therapy [1]. Over the past two decades, the number of deaths caused by pancreatic cancer has doubled to over 441,000 cases globally [2]. Surgery remains the only curative treatment strategy. However, over 80% of PDAC patients are diagnosed with locally advanced or metastatic disease and, therefore, cannot undergo surgery [3]. The 5-year survival rate stands at about 10%, despite advances in management [4,5]. Classic symptoms of PDAC include weight loss, anorexia, abdominal pain, and obstructive jaundice [6]. Some of the risk factors of PDAC include age, obesity, smoking, excessive alcohol intake, chronic pancreatitis (CP), and Type 2 Diabetes Mellitus (T2DM) [7]. Although there is very little biological information on PDAC in the African population, they have been shown to have increased incidence and mortality, attributed to a combination of social (such as excessive smoking and alcohol intake) and genetic factors [8,9,10].

As a hallmark of cancer, tumour cells reprogram their metabolism, such as promoting glycolysis to maintain cell survival and increase proliferation rate [11,12,13]. Metabolites are products of the metabolism that navigate important biological functions such as energy conversion [14,15] and signalling [16,17]. Blood metabolite concentrations can reflect the metabolic adaptation of tumour or highlight the host response to the tumour [18]. In this sense, Nuclear Magnetic Resonance (NMR) spectroscopy was shown to be a powerful technique for the high-throughput analysis of blood samples [19,20]. NMR spectroscopy has been used to investigate the serum metabolome of patients with PDAC to distinguish malignant and benign diseased states and some metabolites, such as leucine, valine, isoleucine, tyrosine, lysine, creatinine, triglycerides, and 3-hydroxybutyrate, were dysregulated [21,22,23,24]. Although these authors reported the blood-based metabolomics biomarkers of PDAC, their findings did not make associations to outcomes and were conducted in other population groups [25,26].

PDAC is a complex and heterogeneous disease. Maladies associated with biological and metabolic processes, such as obstructive jaundice, diabetes, and inflammation, can result in complications that could alter the course of the disease [27]. These maladies could also lead to changes in both metabolic and lipoprotein profiles. For instance, over 70% of PDAC patients, at the time of their diagnosis, presented symptoms of cholestatic jaundice [28], a reduction in or stoppage of bile flow. An abnormal lipoprotein profile has been linked to patients that present with cholestatic jaundice due to the increased bile acid and cholesterol levels [29,30]. T2DM is another common comorbidity that is well known to reflect changes in the serum metabolome. In PDAC, T2DM can promote tumour progression via changes in the transcriptome and metabolome [31]. Its close association with chronic inflammation adds an extra layer to the complexity of this disease [32].

To our knowledge, for the first time, this pilot study shows the links between metabolomic and lipoprotein profiles in PDAC patients of African ancestry with disease stage and patient survival. Additionally, the impact of the metabolic and lipoprotein profile on T2DM, cholestatic jaundice, and inflammation is reported.

## 2. Results

### 2.1. Patients’ Demographic and Clinicopathological Characteristics

Six CP and thirty-four patients with PDAC, including 22 with Resectable Pancreatic Adenocarcinoma (RPC), 8 with Locally Advanced Adenocarcinoma (LAPC) and 4 with Metastatic Adenocarcinoma (MPC), were recruited. Six age-matched healthy controls (HC) were also recruited in this cohort. The demographic features and comorbidities of the patients with PDAC, and CP are reported in Table 1. The demographic features were matched across the four patient groups (i.e., CP, RPC, LAPC and MPC). About 50% (*n* = 21) of the patients are smokers (≥1 packet a day) and 18 patients are alcohol consumers (>100 g of alcohol, which corresponds to six bottles of beer, per day). The frequency of cholestatic jaundice was statistically significant, with a high prevalence in PDAC patients while being absent in all of the CP patients. Of note, five of the PDAC patients developed cholangitis, an inflammation of the bile duct system often caused by bacterial infection, and this was higher in patients with more advanced stages of PDAC. As an expected consequence of cholestatic jaundice, abnormal bilirubin values were observed in PDAC groups compared to the CP group, as reported in Table 2 and shown in Figure S1. Although not statistically significant, the PDAC groups also displayed the typical profile associated with cholestatic jaundice, including increased alkaline phosphatase and gamma-glutamyl transferase activity and a lesser increase in the transaminase enzymes, when compared to the CP patients. Interestingly, T2DM tended to be more frequent amongst CP patients when compared to PDAC patients, although statistical significance was not achieved.

From the routinely collected clinical data, no statistical significance was observed for either routine haematological (Table S1) or chemistry-parameters (Table S2) between the PDAC and CP groups.

### 2.2. Metabolic and Lipoprotein Signatures in the Different Tumour Stages

**Figure 1:**
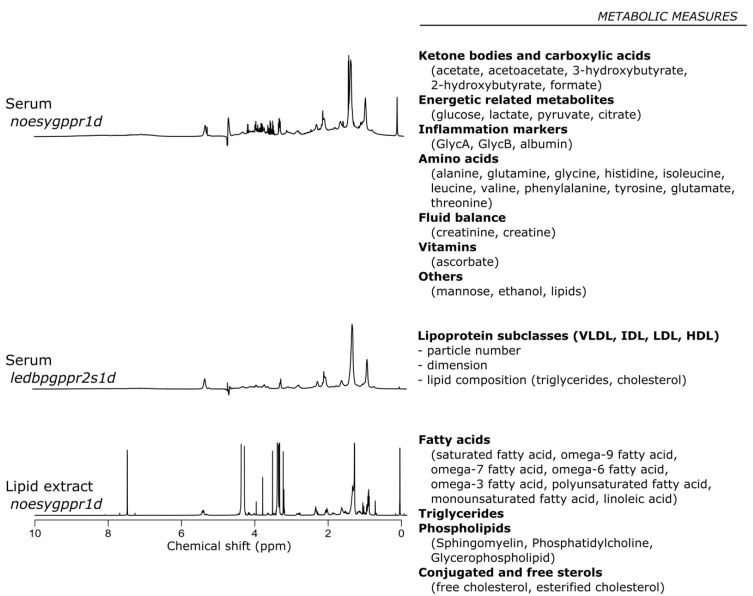
Nuclear Magnetic Resonance experiments showing their relative metabolic measures extracts.

In this study, a serum sample analysis of the cohort was conducted using NMR spectroscopy. Three different sets of NMR experiments were conducted to collect a broad range of information (Figure 1).

**Figure 2:**
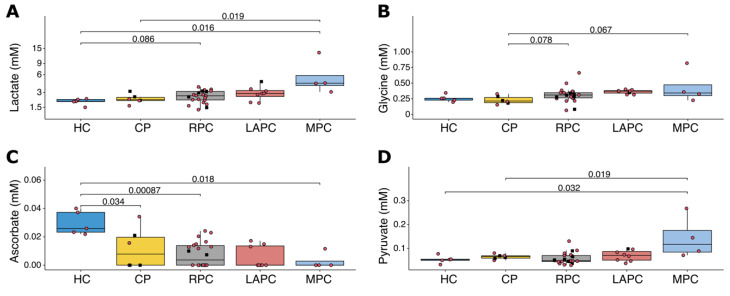
Boxplots showing the comparison of the concentration of (**A**) Lactate, (**B**) Glycine, (**C**) Ascorbate and (**D**) Pyruvate for HC, CP, RPC, LAPC, and MPC. Lactate is shown to be significantly elevated across the groups, glycine was significantly elevated in MPC when compared with CP. Ascorbate was significantly downregulated in CP, RPC, LAPC, and MPC when compared to HC, while pyruvate was significantly upregulated in MPC when compared to HC, CP, HC, RPC, and LAPC. Black squares represent patients with Type 2 Diabetes Mellitus. Red circles represent patients with no Type 2 Diabetes Mellitus. HC: Healthy controls; CP: Chronic Pancreatitis; RPC: Resectable Pancreatic Adenocarcinoma; LAPC: Locally Advanced Pancreatic Adenocarcinoma; MPC: Metastatic Pancreatic Adenocarcinoma.

To delineate the metabolic signatures of PDAC, Spearman correlation’s test was performed to link metabolic values to the different PDAC groups in the following rank order: HC, CP = 1, RPC = 2, LAPC = 3, and MPC = 4. A total of 75 signals were quantified from the NMR spectra of serum samples and lipid extracts (Table S3), including 29 metabolites, 19 lipid classes, inflammatory markers, GlycA and GlycB, and 1 signal that correlated with protein concentration. The analysis of metabolites concentrations in serum samples (Table S4) and lipid extracts (Table S5) revealed that lactate, the end-product of glycolysis under anaerobic conditions, was strongly correlated with the disease stage (rho = 0.50; *p*-value < 0.001; FDR = 0.012). Although not significant, pyruvate, the precursor of lactate, showed a positive correlation with the tumour stage (rho = 0.28, *p*-value = 0.060, FDR = 0.294). Lactate and glucose concentrations were not correlated (rho = 0.06; *p*-value = 0.688). A strong positive correlation with tumour stage was noted with the glycine concentration (rho = 0.52; *p*-value < 0.001, FDR = 0.012). On the other hand, ascorbate (rho = −0.47; *p*-value = 0.001, FDR = 0.021) seems to be depleted or present in a reduced concentration in patients with PDAC. A comparison of the concentrations of lactate, glycine, ascorbate, and pyruvate across the groups HC, CP, RPC, LAPC, and MPC is shown in Figure 2.

A selected number of ratios between metabolite concentrations was selected and associated to one or more enzymatic reaction (Table S6). The analysis of the metabolite ratios (Table S7) showed no association with disease stage. The lipoprotein parameters, including the size, number of particles and concentration of lipids (cholesterol and triglycerides) in the main classes of lipoproteins very-low-density lipoprotein (VLDL), intermediate-density lipoprotein (IDL), low-density lipoprotein (LDL) and high-density lipoprotein (HDL), were estimated using Liposcale test. Negative correlations of some parameters were reported, such as number of HDL particles with disease stage (Table S8). Gamma-glutamyl transferase and the ratio between aspartate transaminase and alanine transaminase were not associated with the disease stage (result not included).

### 2.3. Dysregulated Metabolites in Patient Survival

Wald test, after adjusting for age, was used to identify the metabolites in serum samples as shown in Table S9, the lipid extracts (Table S10), the metabolite ratios (Table S11), and the lipoprotein parameters (Table S12) that correlated with the time of survival. Both 3-hydroxybutyrate (*p*-value = 0.015; FDR = 0.370) and ethanol (*p*-value = 0.002; FDR = 0.126) were independently correlated with the survival time in patients with PDAC. Cox hazard analysis showed that a statistically significant higher hazard ratio (HR) exists between the patients with the 20% highest concentration of ethanol compared to the rest (HR = 4.22 [95%CI: 1.44–12.32]; *p*-value = 0.009) and between the patients with 20% highest concentration of 3-hydroxybutyrate compared to the rest (HR = 2.88 [95%CI: 1.02–8.11]; *p*-value = 0.045). Gamma-glutamyl transferase and the ratio between aspartate transaminase and alanine transaminase was not associated with the survival time (result not included).

**Figure 3:**
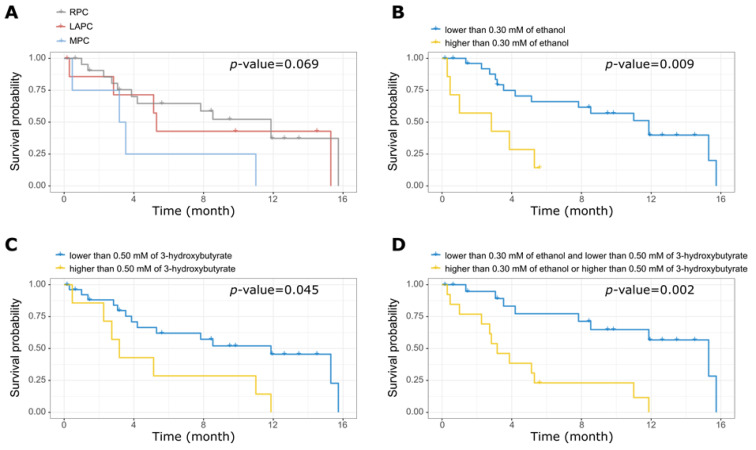
Impact of tumour stages and metabolites concentration on patient survival. Kaplan–Meier survival curves showing effect of (**A**) Tumour stage, (**B**) Ethanol, (**C**) 3-hydroxybutyrate, and (**D**) combination of ethanol and 3-hydroxybutyrate with survival time. There was no significant link between tumour stages and patient survival. PDAC patients with low levels of both ethanol and 3-hydroxybutyrate survived longer. RPC: Resectable Pancreatic Adenocarcinoma; LAPC: Locally Advanced Pancreatic Adenocarcinoma; MPC: Metastatic Pancreatic Adenocarcinoma.

Patients with 20% highest concentrations of ethanol and 3-hydroxybutyrate were grouped. This combined group showed significantly poorer survival than the remaining patients (HR = 5.87 [95%CI: 1.92–17.92]; *p*-value = 0.002). No correlation was observed between PDAC stages, lipid extracts, metabolite and lipoprotein levels, and survival time. Figure 3 shows Kaplan–Meier plots of the survival time, segregated according to the value of ethanol, 3-hydroxybutyrate, and a combination of them, as described earlier.

### 2.4. Impact of Raised Bilirubin Levels on Metabolites and Lipoproteins in PDAC

**Figure 4:**
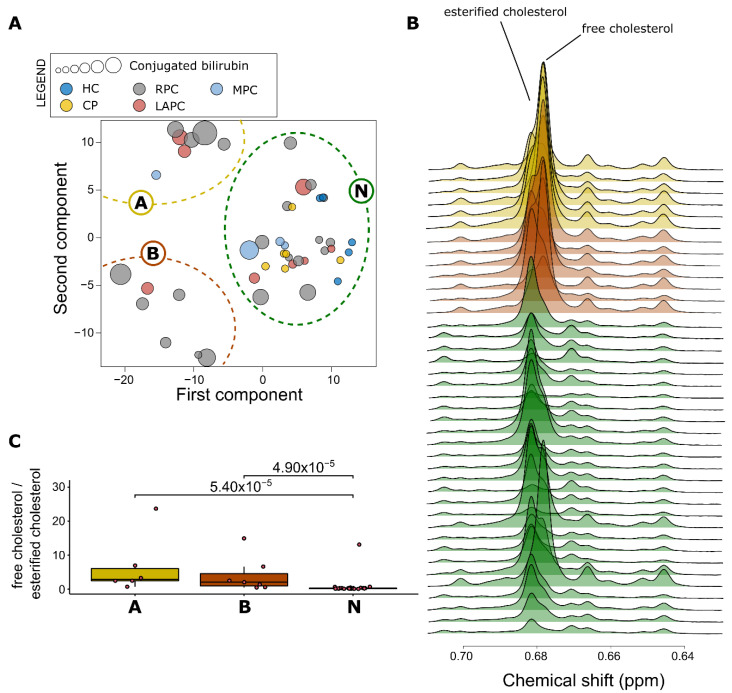
Measurement of Lipoprotein concentration in patient groups. (**A**) Lipoprotein particle concentrations were measured from the NMR spectra using LipoScale test and were separated into 3 clusters. Cluster N, which is made up of all the controls and some PDAC patients, has a normal lipoprotein profile, while clusters A and B have atypical lipoprotein profiles with high bilirubin levels. (**B**) Spectra showing the abnormal lipoprotein profile associated with clusters A (yellow) and B (red) with a high concentration of free cholesterol, which could be indicative of an abnormal lipoprotein profile while cluster N (green) all have a normal lipoprotein profile; higher levels of esterified cholesterol, except for one outlier. (**C**) Boxplot of the ratio of free cholesterol to esterified cholesterol was calculated for the three clusters to comprehend the level of lipoprotein abnormality in the serum. Cluster N has the least ratio. PDAC: Pancreatic Ductal Adenocarcinoma.

PAM clustering was performed on the KODAMA scores to identify any distinct lipoprotein phenotype (Figure 4A). Three distinct clusters were identified. All HC and CP patients were classified in the largest cluster (N). The lipoprotein parameters of the individuals classified in the cluster N showed values similar to those of the general population [33]. The patients classified in one of the other two clusters (A and B) showed an atypical lipoprotein profile. To verify if patients belonging to clusters A or B showed signs of cholestatic jaundice, as suggested by Lamiquiz-Moneo et al. [34], the concentration of cholesterol ester and free cholesterol was evaluated in the lipid extracts (Figure 4B). The ratio between free cholesterol and cholesterol ester was used as a marker to identify the presence of an abnormal lipoprotein produced in patients with cholestatic jaundice [29]. Figure 4C shows that patients belonging to the clusters A and B have a higher ratio indicative of the possible presence of abnormal lipoprotein. Using the ratio between free cholesterol and cholesterol ester, a threshold of 0.45 was identified to discriminate clusters A and B from cluster N. All patients belonging to clusters A and B had values above 0.45. All patients belonging to cluster N had values below 0.45, except for three patients.

Supervised PLS analysis was then performed to identify variance in the metabolic profiles of DIFF spectra associated with the ratio between free cholesterol and cholesterol ester; the resulting model demonstrated a clear and robust discrimination between patient’s ratio values below and above 0.45 (R2 = 0.81, 95% CI 0.81–0.86; Q2 = 0.70, 95% CI 0.69–0.72; *p*-value < 0.001). The cross-validated model was able to discriminate the two groups with an accuracy of 90%, a sensitivity of 93.75%, and a specificity of 87.50%.

Both A and B clusters showed atypical lipoprotein expression and were then grouped as “AB”, to understand the effects of the altered ratio (free cholesterol/cholesterol ester) in lipoproteins, a comparison of the N versus AB clusters was performed for full blood count features (Table S13), blood chemistry features (Table S14) and liver function parameters (Table S15). As expected, most of the liver function parameters were significantly altered; total bilirubin (*p*-value = 0.003, FDR = 0.012), conjugated bilirubin (*p*-value = 0.006, FDR = 0.016) and aspartate transaminase (*p*-value = 0.009, FDR = 0.018) increased in clusters AB. Furthermore, some metabolites (Table S16), such as total protein (*p*-value < 0.001, FDR < 0.001), glutamine (*p*-value < 0.001, FDR = 0.007) reduced in concentration, whereas lipid levels (*p*-value < 0.001, FDR = 0.001) were elevated in clusters AB. Lipid extracts (Table S17), metabolite ratios (Table S18) and lipoproteins (Table S19) were significantly altered.

### 2.5. Impact of Diabetes and Inflammation on Metabolites and Lipoproteins Levels

In order to determine the impact of diabetes and inflammation on the metabolic signatures of PDAC patients and their link with the tumour stages, the serum metabolite concentrations between patients with and without T2DM were compared using Wilcoxon rank-sum test. With regards to patients with T2DM, no statistically significant difference between the CP and PDAC groups in the metabolite and lipoprotein concentrations was detected (results not included).

**Figure 5:**
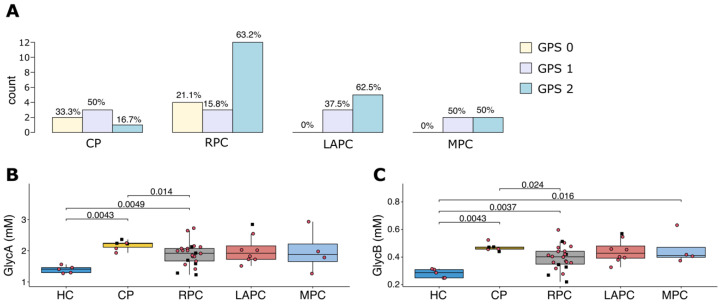
Inflammation status in patient groups (**A**) shows the inflammation levels of PDAC and CP groups using Glasgow Prognostic Score (GPS), CP (6) has the least inflammation and PDAC (RPC:22; LAPC: 8; MPC: 4.) groups are all highly inflamed. GlycA (**B**) and GlycB (**C**) show the comparison of the inflammatory status of PDAC and control groups (HC and CP) using GlycA and GlycB biomarkers, respectively. There was no difference observed across the groups for both GlycA and GlycB levels except when compared with the HC. The black square boxes represent T2DM patients. Red circles represent patients with no T2DM. T2DM: Type 2 Diabetes Mellitus; PDAC: Pancreatic Ductal Adenocarcinoma; HC: Healthy controls; CP: Chronic Pancreatitis; RPC: Resectable Pancreatic Adenocarcinoma; LAPC: Locally Advanced Pancreatic Adenocarcinoma; MPC: Metastatic Pancreatic Adenocarcinoma.

Then, the inflammatory status of the patients using both the Glasgow Prognostic Score (GPS) and the NMR inflammatory biomarkers, GlycA and GlycB were compared (Figure 5). GPS is a cumulative inflammation-based cancer prognostic marker based on elevated serum CRP and decreased albumin concentration [35]. The percentage of patients with GPS = 2 is higher in PDAC than CP. The NMR inflammatory marker GlycA and GlycB were lower in HC compared to the pathology groups (Figure 5B,C). Slightly higher values of GlycA and GlycB were observed in CP compared to PDAC. Over 14% of the PDAC patients had cholangitis and showed only slightly higher values of CRP (*p*-value = 0.064), which were not significant.

However, we identified metabolites (Table S20) such as glucose, lactate, histidine, phosphorous, lipid extracts (Table S21), glucose/lactate, threonine/glycine ratios (Table S22) and lipoproteins (Table S23) that correlated with inflammatory markers: GlycA, GlycB, CRP, and Albumin. Glucose was shown to correlate directly with GlycB (Figure S2).

## 3. Discussion

PDAC has an almost equal number of new cases and deaths annually. Hence, there is a need for further investigation of underlying molecular underpinnings, especially in under-studied patient groups. Although patients of African descent have an elevated risk and poor survival rates of PDAC, there is little molecular and clinical information for this group. The combination of an analysis of metabolites and lipoproteins profiles with clinical parameters may improve management decisions and outcomes [36]. In this study, preliminary data showing metabolomic and lipoprotein perturbations in our patient group were observed at different stages of PDAC.

As the severity of PDAC increased from resectable to metastatic, there was an observed elevation of levels of lactate and glycine and reduced levels of ascorbate. Thus, these metabolite profiles could help to distinguish PDAC severity. In PDAC cells, there is an increased uptake of glucose to produce lactate and ATP under aerobic conditions, a phenomenon known as the Warburg effect [37]. Warburg effect promotes PDAC progression by providing a constant energy source for cellular growth and proliferation. Additionally, enhanced glycolysis leads to the generation of substrates such as pyruvate and, subsequently, lactate, which promote tumour growth [38]. Furthermore, the correlation between glucose/lactate ratio with CRP, which was observed in this study, could suggest that glycolysis is elevated with inflammation.

Glycine is formed from 3-phosphoglycerate an intermediate of glycolysis pathway [39], thus upregulated glycolysis could result in elevated glycine levels. Glycine is also the main substrate in glutathione and collagen production [40,41], which are essential in PDAC progression. The activation of serine/glycine biosynthesis promotes tumorigenesis by delivering a single carbon for 1-carbon metabolism of proteins, lipids, nucleic acids, and other biological macromolecules to support tumour growth [42]. Furthermore, this study observed that the Threonine/Glycine ratio has a direct association with albumin, inferring an increase in glycine levels with inflammation.

This study also showed a link between reduced levels of ascorbate (vitamin C) and the severity of PDAC. Oxidized ascorbate (dehydroascorbate) is transported into cells via glucose transporters, after which it is reduced to ascorbate using glutathione [43]. It acts as a pro-oxidant triggering reactive oxygen species activities, which inhibit a key glycolytic enzyme, Glyceraldehyde-3-Phosphate Dehydrogenase (GAPDH), in cancer cells [44]. Reduced levels of ascorbate may imply a deregulation of the glycolysis rate, resulting in the Warburg effect, which, in turn, may favour PDAC progression. This hypothesis is supported by various studies that have identified the therapeutic roles of ascorbate in PDAC. A combination of ascorbate and gemcitabine achieved a more significant tumour growth inhibition in the mouse model than gemcitabine alone; additionally, pharmaceutic doses of vitamin C act as a pro-oxidant and reduce tumour growth in mice xenografts [45,46]. The anti-tumour effect was further observed when ascorbate inhibited epithelial-to-mesenchymal transition and, consequently, metastasis in both in vitro and in vivo models [45]. The administration of ascorbate was also demonstrated to improve survival in a stage IV PDAC patient with little toxicity observed [47].

Interestingly, this study showed that ethanol and 3-hydroxybutyrate (3-HB) have a negative correlation with survival time and are independent of the disease stage. Several studies have contradictory results on the role of 3-hydroxybutyrate in pancreatic cancer [24,26]. PDAC cells adapt their metabolism to the environment they are exposed to by utilizing the diverse fuels that are available [48]. Excessive amounts of ketone bodies are usually found in individuals with diabetic ketoacidosis (DKA) or alcoholic ketoacidosis (AKA) [49]. This study suggests that high 3-HB levels could be linked to alcohol consumption or T2DM and not necessarily to the pathology. In DKA, a lack of insulin contributes to ketogenesis in the liver. DKA is also linked to an altered ratio of 3-HB to acetoacetate [49], although there was no association with staging and survival in our cohort. One study showed that heavy alcohol consumption was a contributing risk factor of PDAC, especially in black women [8]. Indeed, it is well-known that high concentrations of ethanol inhibit lipolysis, while a substantial production of ketone bodies such as 3-hydroxybutyrate occurs with its decrease [50]. In liver cells, AKA causes a change in redox potential induced by alcohol and reduces oxaloacetate levels [50]. Although the mechanism leading to early deaths in PDAC patients who are alcohol consumers is unclear, one theory is that the use of 3-hydroxybutyrate by oxidative mitochondrial metabolism can induce the proliferation and migration of cancer cells [51,52]. In addition, ascorbate depletion in PDAC patients due to heavy alcohol consumption could both increase glycolysis rate, thereby promoting the severity of the disease and inhibit glycogen synthesis in the tumour microenvironment [53]. The analysis of the metabolic profile could be used to understand the potential role of alcohol consumption in predicting patient outcomes.

Most of the PDAC patients in this study have elevated bilirubin levels, reflecting an obstruction in the bile duct by the tumour. Clinically, cholestatic jaundice can be diagnosed when the ratio of total bilirubin to conjugated bilirubin is greater than 50% and there are elevated levels of other clinical liver parameters, such as alkaline phosphatase (ALP) and gamma-glutamyl transferase (GGT) [54]. Since these parameters are used to assess liver function, they can also be linked to liver diseases or injury [55]. Furthermore, chronic inflammation induces a variety of alterations in lipid metabolism, which are accompanied by an altered ratio of free cholesterol to cholesterol ester and associated with an abnormal lipoprotein profile [29]. This study confirms the previously reported association between an atypical lipoprotein profile with cholestatic jaundice [34], suggesting that the detection of abnormal lipoprotein profile might be used to identify cholestatic jaundice in PDAC patients. We hypothesize that altered lipid metabolism observed in PDAC patients [56] could be due to the effects of cholestatic jaundice in these patients. In the clinical setting, analysis of the lipoprotein profile could be used to understand the need for a stent placement to relieve obstruction and evaluate the success of this treatment.

Although inflammation has emerged as an important player in pancreatic cancer development and progression [57], NMR inflammatory markers, GlycA and GlycB, were not able to discriminate between CP and PDAC. Although few PDAC patients developed cholangitis, which is an inflammation of the biliary tract, they presented a generally high level of inflammation, as expected. GlycA and GlycB were not able to stratify the patients based on the tumour staging. However, a fingerprint of the inflammatory processes was observed in the metabolic profile. Interestingly, the positive correlation between GlycB and glucose concentration could enrich the long-standing debate on the link between inflammation and diabetes. The higher level of glucose detected in the blood could be due to the effect of chronic inflammation on decreasing insulin secretion and sensitivity [58].

Despite some of the statistically significant data, the small number of recruited patients in each stage might be a limitation. Furthermore, although several findings were identified, the study is descriptive in nature. This pilot study is part of an ongoing project and a future study would aim to validate these findings in a larger patient cohort.

## 4. Materials and Methods

### 4.1. Sample Collection and Processing

The study was approved by the University of Witwatersrand Human Research Ethics Committee (Medical) (Study number—M190681). All participants gave written informed consent. Patient clinical data were collected using the REDCap v9.0 [59]. Sample and data collection were conducted between March 2019 and March 2020. The study site was the Hepatopancreatobiliary Unit at Chris Hani Baragwanath Academic Hospital, Soweto Johannesburg, South Africa.

Only patients with clinically and histologically proven PDAC were recruited for this study. Inclusion criteria included patients from 18 years old and above, of African ancestry, and diagnosed with one of the three stages of PDAC. African ancestry in this cohort are black patients who self-reported to belong to one of the ethnic groups of South Africa. Patients undergoing chemotherapy at the time of the study were excluded. Stratification into resectable, borderline resectable, locally advanced, and metastatic disease was conducted with a contrast-enhanced triple-phased CT-scan of the abdomen following the National Comprehensive Cancer Network (NCCN) guidelines [60]. For this study, both resectable and borderline resectable were categorized as RPC. In this group, the tumour either had not invaded any vessel or had invaded the portal vein to 90°, in which case neoadjuvant chemotherapy may be necessary before surgery. The LAPC group included cases where the tumour had invaded the superior mesenteric artery and/or portal vein to more than 180°. Lastly, in the MPC, the tumour had spread to other organs such as the liver [61]. CP patients and HC patients, also of African ancestry, were recruited as the control arm of the study. To be eligible, all the healthy participants confirmed that they were in good health and were not taking any regular medication. Blood samples were collected during fasting by venepuncture in clear vacutainer tubes (BD Biosciences, Franklin Lakes, NJ, USA) without anti-coagulant. The blood was centrifuged at 3000 rpm, 4 °C for 10 min after allowing it to clot for 30–60 min at room temperature. All samples were processed within 2 h of collection and immediately stored at −80 °C until analysis.

### 4.2. Serum Sample Preparation

Three hundred microliters of thawed serum samples were aliquoted into a microcentrifuge tube and followed by 300 µL of a solution containing 0.75 M potassium phosphate buffer (pH 7.4), 5.81 mM of trimethylsilyl-2,2,3,3-tetradeuteropropionic acid (TSP; Sigma-Aldrich, St. Louis, MO, USA) and a trace amount of sodium azide dissolved in deuterium oxide. Samples were vortexed to ensure complete homogeneity and a final volume of 540 µL of each sample was transferred to a 5 mm NMR tube (Wilmad Lab Glass, Vineland, NJ, USA) and analysed.

### 4.3. Lipid Extracts Preparation

Lipids were extracted using the protocol described by Lamiquiz Moneo et al. [34]. Three hundred microliters of BUME (butanol:methanol—2:1) was added to a 100 µL serum aliquots in glass GC vials followed by 300 µL DIPE (diisopropyl ether:ethyl acetate—3:1) and 300 µL H_2_O. Samples were vortexed for one minute after addition of BUME and H_2_O and incubated on a shaker for 10 min after DIPE addition to allow for lipid extraction. The samples were then centrifuged at 4000 rpm for 5 min, after which the top layer was transferred to clean vials, dried under N_2_ at 37 °C and then resuspended in 600 µL solution of CDCl_3_:CD_3_OD:D_2_O (chloroform-d:methanol-d:water-d; 16:7:1, *v*/*v*/*v*) containing 1.18 mMTSP. Five hundred and forty microlitres of this final solution was then transferred to 5 mm NMR tube (Wilmad Lab Glass) for analyses.

### 4.4. Nuclear Magnetic Resonance Spectroscopic Analysis

One-dimensional (1D) proton (^1^H)-NMR spectra was acquired using different pulse sequences on a 500 MHz Bruker Avance III HD NMR spectrometer equipped with a triple-resonance inverse 1H probe head and x, y, z gradient coils. A standard nuclear overhauser effect spectroscopy (NOESY) pulse sequence presat (noesygppr1d.comp) was used on both serum and lipid extract samples. On serum samples, NOESY was used to detect both signals of small metabolites and high-molecular-weight macromolecules such as lipoproteins. Additionally, a standard diffusion-edited (DIFF) pulse sequence (ledbpgppr2s1d) was used on serum samples to detect only high-molecular-weight macromolecules, such as lipoproteins. Pooled samples were used as a quality control sample and were included in each batch for qualitative assessment of repeatability by overlaying the raw spectra.

### 4.5. Nuclear Magnetic Resonance Profiling

NMR spectroscopy was used to quantify a panel of 75 signals. The peaks of the identified metabolites were fitted by combining a local baseline and Voigt functions based on the multiplicity of the NMR signal [62]. The assignment of quantified signals is reported in Table S3. To validate the efficacy of the different deconvolution models, the root-mean-square deviation was determined. The absolute concentration of each metabolite was calculated according to the previously reported equation [63]. The number of protons contributing to the unknown signals was imputed to 1. The concentration of carbohydrates was also estimated by considering the equilibrium between their cyclic forms.

GlycA and GlycB signals were quantified by integrating the areas between 2.00 and 2.05 ppm and between 2.09 and 2.05 ppm, respectively, above a local baseline, aiming to remove the signal of lipoproteins. The Liposcale test (Biosfer TesLab, Reus, Spain) was then used to determine lipoprotein parameters, HDL, LDL, and VLDL particle number, size, and lipid concentration of each subtype [64]. Each DIFF spectrum in the range between 0.1 and 9.5 ppm, excluding the regions corresponding to the water signals between 4.40 and 5.00 ppm, was segmented into 0.001-ppm chemical shift bins, and the corresponding spectral areas under the curve, giving a total of 8800 variables.

### 4.6. Statistic and Data Analysis

Statistical analysis and graphical illustrations of the data were generated in R (version 3.6.1) and R studio (version 1.1.456) software using scripts developed in-house. Wilcoxon and Kruskal–Wallis rank-sum test was used to compare differences in numerical covariates (e.g., age and metabolite concentration). Fisher’s exact test was used to assess differences between categorical variables (e.g., gender). Spearman’s rank test was used to calculate the correlation coefficient (rho) between variables. The Wald test was used to calculate the statistical significance (*p*-value) of the differences between the Kaplan–Meier survival curves. Prognostic factors for overall survival were analysed using the Cox proportional hazard regression. *p*-values < 0.05 were considered significant. To account for multiple testing, a false discovery rate (FDR) of <10% was applied.

The KODAMA algorithm [65] was used to identify of patterns that demonstrate metabolic phenotypes across all samples [66]. Using the partition around medoids (PAM) clustering [67] was applied to the KODAMA scores using the silhouette algorithm 10 [68] to verify the obtained results. The silhouette median value was utilized to assess the ideal number of clusters, ranging from 2 to 10.

Using partial least-squares (PLS) analysis, regression was performed on DIFF spectra metabolic profiles. Furthermore, a 10-fold cross-validation was performed to evaluate the predictive efficacy of the model [19]. Both the goodness of fit parameter (R^2^) and the predictive ability parameter (Q^2^) were also calculated using standard formulas [69]. The Q^2^ value was calculated from *p*-value to assess the performance of the PLS regression model [70]. A *p*-value < 0.05 was regarded as significant.

## 5. Conclusions

In our cohort, we demonstrated that obstructive jaundice, T2DM, and inflammation can contribute to defining the metabolic phenotype in PDAC, thus evaluating that their patterns could help to predict prognosis, whereby patients at high risk of late-stage disease may benefit from better management decisions. The depletion of vitamin C in PDAC patients with a high alcohol consumption rate reiterates its therapeutic role. Furthermore, evaluating the lipoprotein profiles in patients could help to more accurately identify those with obstructive jaundice that may require urgent treatment; however, this has to be verified.

## Figures and Tables

**Table 1:** Demographic features and clinicopathological of Chronic Pancreatitis and Pancreatic Ductal Adenocarcinoma patients.

Feature	HC (*n* = 6)	CP (*n* = 6)	RPC (*n* = 22)	LAPC (*n* = 8)	MPC (*n* = 4)	*p*-Value
HIV status						0.831
Negative, *n* (%)	6 (100.0)	5 (83.3)	19 (86.4)	6 (75.0)	4 (100.0)	
Positive, *n* (%)	0 (0.0)	1 (16.7)	3 (13.6)	2 (25.0)	0 (0.0)	
Gender						0.286
female, *n* (%)	3 (50.0)	0 (0.0)	8 (36.4)	2 (25.0)	2 (50.0)	
male, *n* (%)	3 (50.0)	6 (100.0)	14 (63.6)	6 (75.0)	2 (50.0)	
Smoking						0.450
no, *n* (%)	6 (100.0)	1 (16.7)	12 (54.5)	4 (50.0)	2 (50.0)	
yes, *n* (%)	0 (0.0)	5 (83.3)	10 (45.5)	4 (50.0)	2 (50.0)	
Alcohol						0.962
no, *n* (%)	4 (66.67)	3 (50.0)	13 (59.1)	4 (50.0)	2 (50.0)	
yes, *n* (%)	2 (33.33)	3 (50.0)	9 (40.9)	4 (50.0)	2 (50.0)	
Age, median (IQR)	37 (24–54)	51 (46–57)	63 (50–67)	56 (48–62)	56 (46–70)	0.439
Obstructive jaundice						0.013
no, *n* (%)	6 (100.0)	6 (100.0)	8 (36.4)	2 (25.0)	1 (25.0)	
yes, *n* (%)	0 (0.0)	0 (0.0)	14 (63.6)	6 (75.0)	3 (75.0)	
Cholangitis						0.145
no, *n* (%)	6 (100.0)	6 (100.0)	20 (90.9)	7 (87.5)	2 (50.0)	
yes, *n* (%)	0 (0.0)	0 (0.0)	2 (9.1)	1 (12.5)	2 (50.0)	
T2DM						0.322
no, *n* (%)	6 (100.0)	3 (50.0)	16 (72.7)	7 (87.5)	4 (100.0)	
yes, *n* (%)	0 (0.0)	3 (50.0)	6 (27.3)	1 (12.5)	0 (0.0)	
Hypertension						0.560
no, *n* (%)	6 (100.0)	6 (100.0)	17 (77.3)	6 (75.0)	4 (100.0)	
yes, *n* (%)	0 (0.0)	0 (0.0)	5 (22.7)	2 (25.0)	0 (0.0)	

Healthy Controls (HC) are not included in statistical analysis IQR: interquartile range; T2DM: Type 2 Diabetes Mellitus; CP: chronic pancreatitis; RPC: Resectable Pancreatic Ductal Adenocarcinoma; LAPC: Locally Advanced Pancreatic Ductal Adenocarcinoma; MPC: Metastatic Pancreatic Ductal Adenocarcinoma.

**Table 2:** Liver Function Tests of the Chronic Pancreatitis and Pancreatic Ductal Adenocarcinoma groups.

Feature	* Physiological Range	CP Median	RPC Median	LAPC Median	MPC Median	*p*-Value	FDR
Total Protein (g/L)	60–78	66.0	59.0	66.0	69.0	0.289	0.330
Albumin (g/L)	35–52	36.5	30.0	27.0	32.5	0.361	0.361
Total Bilirubin (µmol/L)	5–21	5.0	154.0	120.0	58.0	0.006	0.030
Conjugated Bilirubin (µmol/L)	0–3	2.0	141.0	112.5	45.0	0.008	0.030
Alanine transaminase (U/L)	10–40	18.0	88.0	29.5	38.0	0.051	0.082
Aspartate transaminase (U/L)	15–40	28.5	104.0	55.0	75.0	0.019	0.052
Alkaline phosphatase (U/L)	53–128	74.0	615.0	337.0	314.5	0.025	0.052
Gamma glutamyl transferase (U/L)	<68	61.5	751.0	301.0	483.0	0.151	0.201

FDR: false discovery rate; CP: chronic pancreatitis; RPC: Resectable Pancreatic Ductal Adenocarcinoma; LAPC: Locally Advanced Pancreatic Ductal Adenocarcinoma; MPC: Metastatic Pancreatic Ductal Adenocarcinoma. * Physiological range calculated by Bio Analytical Research Corporation South Africa (https://www.barcsa.co.za/test-directory/test-reference-ranges/, accessed on 6 September 2021).

## Data Availability

The data presented in this study are available in the article and Supplementary Materials.

## Supplementary Materials

The following are available online at https://www.mdpi.com/article/10.3390/metabo11100663/s1, Figure S1: Boxplot of Total Bilirubin across all the groups with *p*-values, Figure S2: Correlation of Glucose and Inflammatory markers, Table S1: Haematological parameters of the Pancreatic Ductal Adenocarcinoma and Chronic Pancreatitis patient groups, Table S2: Blood chemistry of the Pancreatic Ductal Adenocarcinoma and Chronic Pancreatitis patient groups, Table S3: List of the quantified signal and their relative assignment and multiplicity, Table S4: Correlation of concentration of metabolites with the stages of Pancreatic Ductal Adenocarcinoma, Table S5: Correlation of concentration of lipid extracts with the stages of Pancreatic Ductal Adenocarcinoma, Table S6: Selected metabolites ratios and their catalysing enzymes, Table S7: Correlation of metabolites ratios with the stages of Pancreatic Ductal Adenocarcinoma, Table S8: Correlation of concentration of lipoproteins with the stages of Pancreatic Ductal Adenocarcinoma, Table S9: Correlation of metabolites with survival time, Table S10: Correlation of the lipid extracts with survival time, Table S11: Correlation of metabolite concentration ratios with survival time, Table S12: Correlation of lipoproteins with survival time, Table S13: Comparison of the Full Blood Count of N versus AB Clusters, Table S14: Comparison of the Blood chemistry of N versus AB components, Table S15: Comparison of the Liver function tests of N versus AB components, Table S16: Comparison of the metabolite concentration of N versus AB clusters, Table S17: Comparison of the lipid extracts concentration of N versus AB clusters, Table S18: Comparison of the metabolite concentration ratios of N versus AB clusters, Table S19: Comparison of the lipoprotein profile for N versus AB clusters, Table S20: Correlation of metabolites with GlycA, GlycB, CRP, and Albumin intensity value, Table S21: Correlation of lipid extracts with GlycA, GlycB, CRP, and Albumin intensity value, Table S22: Correlation of metabolite concentration ratios with GlycA, GlycB, CRP, and Albumin intensity value, Table S23: Correlation of lipoproteins with GlycA, GlycB, CRP, and Albumin intensity value.
