# Supplementary material for: Serum Metabolomic and Lipoprotein Profiling of Pancreatic Ductal Adenocarcinoma Patients of African Ancestry
Source: Metabolites. 2021 Sep 28;11(10):663. doi: 10.3390/metabo11100663 (PMC8540259; doi:10.3390/metabo11100663)
Supplement: Supplementary file 1 [file metabolites-11-00663-s001.zip › metabolites-1355105-SI.pdf]

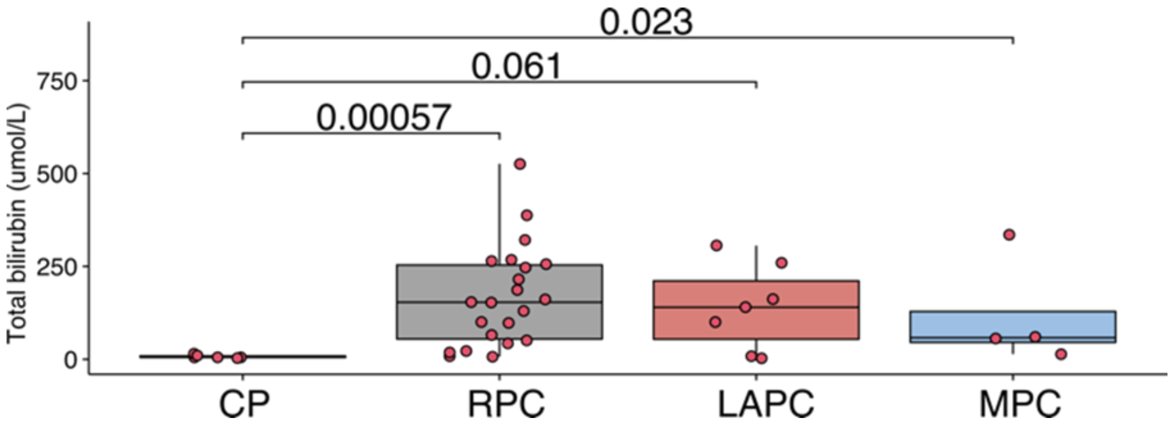

Figure S1: **Boxplot of Total Bilirubin across all the groups with p-values**. Showing statistically significant higher values of Total Bilirubin in PDAC groups RPC, LAPC and MPC compared to CP using Wilcoxon Rank Sum test. Higher values of bilirubin may be due by bile obstruction, which might cause damage to the liver. CP: Chronic pancreatitis; RPC: Resectable Pancreatic Ductal Adenocarcinoma; LAPC: Locally Advanced Pancreatic Ductal Adenocarcinoma; MPC: Metastatic Pancreatic Ductal Adenocarcinoma

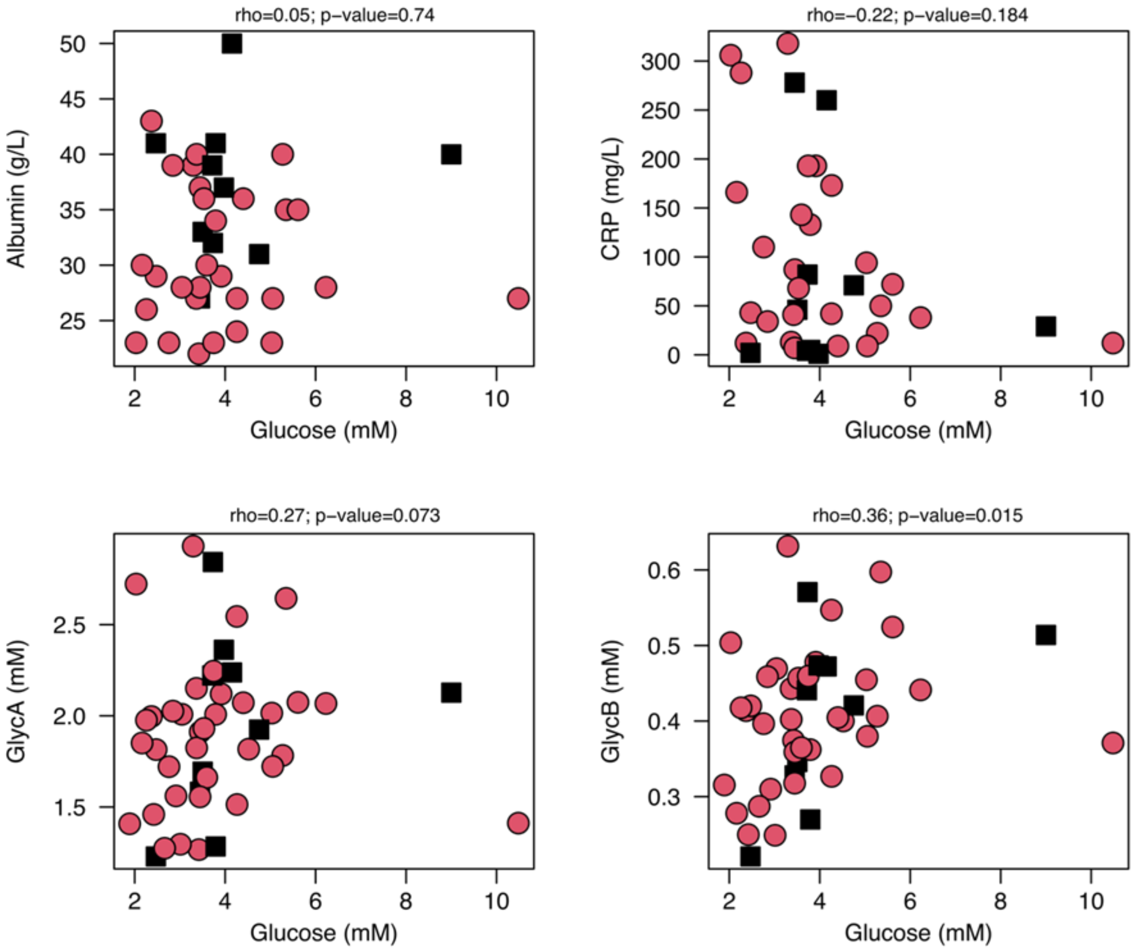

Figure S2: **Correlation of Glucose and Inflammatory markers.** GlycB has a positive correlation with glucose. Elevated glucose levels detected in the blood could be because of chronic inflammation on decreasing insulin secretion and sensitivity. Black-square boxes represent PDAC patients with T2DM. There was no direct correlation of diabetes with the markers. The outlier could be sample collected from a patient with high blood sugar levels after feeding. T2DM: Type 2 Diabetes, PDAC: Pancreatic Ductal Adenocarcinoma.

Table S1: Haematological parameters of the Pancreatic Ductal Adenocarcinoma and Chronic Pancreatitis patient groups.

Feature	CP median [IQR]	RPC median [IQR]	LAPC median [IQR]	MPC median [IQR]	p-value	FDR
Haematocrit	0.385 [0.282 0.455]	0.316 [0.281 0.367]	0.303 [0.244 0.325]	0.339 [0.304 0.373]	0.482	0.620
White cell count (/L^9)	6.83 [6.153 8.182]	8.89 [6.91 12.4]	10.845 [7.927 11.037]	9.905 [4.225 17.31]	0.484	0.620
Haemaglobin (g/DL)	12.7 [9.6 14.6]	10.25 [9.475 11.525]	9.4 [7.825 10.775]	10.95 [10 11.6]	0.507	0.620
Platelet count (/L^9)	245 [222.75 339.25]	372.5 [313 502.75]	436.5 [324.25 497]	358 [317.75 405.25]	0.206	0.594
Prothrombin time	12.1 [11.675 12.9]	13.2 [12.5 15.2]	14.35 [13.025 14.525]	14.2 [13.525 16.05]	0.151	0.594
INR	0.935 [0.927 0.975]	1.08 [1 1.175]	1.165 [1.048 1.183]	1.165 [1.122 1.317]	0.038	0.414
MCV (fL)	92.25 [88.525 93.275]	93 [88 98.3]	89.45 [87.675 91.675]	91.25 [86.75 95.15]	0.647	0.684
MCH (pg)	30.05 [28.25 31.325]	30.2 [28.9 32.5]	29.95 [28.825 30.825]	28.5 [27.85 29.2]	0.260	0.594
MCHC (g/dl)	32.3 [31.75 33.15]	33.4 [32.2 33.8]	33.1 [31.725 33.575]	31.05 [30.8 31.775]	0.312	0.594
MPV (fL)	10.3 [9.2 10.5]	10.5 [9.6 10.9]	10.4 [9.675 11.2]	10.9 [10.575 11.225]	0.324	0.594
Red cell count (/L^12)	4.09 [3.018 4.878]	3.49 [2.99 3.83]	3.325 [2.538 3.852]	3.77 [3.425 4.095]	0.684	0.684

CP: chronic pancreatitis; RPC: Resectable Pancreatic Ductal Adenocarcinoma; LAPC: Locally Advanced Pancreatic Ductal Adenocarcinoma; MPC: Metastatic Pancreatic Ductal Adenocarcinoma

Table S2: Blood chemistry of the Pancreatic Ductal Adenocarcinoma and Chronic Pancreatitis patient groups.

**Features**	**CP median [IQR]**	**RPC median [IQR]**	**LAPC median [IQR]**	**MPC median [IQR]**	**p-value**	**FDR**
Sodium (mmol/L)	138 [135.5 139.75]	137 [135 140]	135.5 [129.5 137.5]	139.5 [138.5 141]	0.295	0.889
Potassium (mmol/L)	4.05 [3.7 4.4]	3.7 [3.4 4.1]	3.55 [3.275 4.075]	3.7 [3.125 4.3]	0.659	0.889
Chloride (mmol/L)	97.5 [97 101.75]	96 [93 97]	93.5 [89.75 96.5]	90.5 [88.75 93]	0.127	0.889
Bicarbonate (mmol/L)	19.5 [18.25 20.75]	23 [17 25]	20 [19.75 21.5]	24.5 [18.5 30.25]	0.800	0.889
Anion gap (mmol/L)	24.5 [20.75 26.75]	26 [19 30]	23 [22 26.25]	29.5 [25.75 33]	0.445	0.889
Calcium (mmol/L)	2.26 [2.198 2.382]	2.2 [2.11 2.31]	2.105 [1.978 2.235]	2.135 [2.128 2.148]	0.403	0.889
Magnesium (mmol/L)	0.865 [0.83 0.885]	0.79 [0.68 0.9]	0.77 [0.682 0.885]	0.78 [0.72 1.16]	0.800	0.889
Inorganic Phosphate (mmol/L)	1.18 [1.13 1.32]	1.06 [0.96 1.2]	1.085 [0.995 1.278]	1.14 [1.098 1.367]	0.477	0.889
Urea (mmol/L)	4 [3.45 5.45]	4 [2.2 5.5]	3.5 [2.075 7.125]	4.35 [3.05 6.325]	0.945	0.645
Creatinine (µmol/L)	90 [64.25 130]	71 [58 95]	64.5 [48 79.75]	76.5 [72.75 80.25]	0.585	0.889

CP: chronic pancreatitis; RPC: Resectable Pancreatic Ductal Adenocarcinoma; LAPC: Locally Advanced Pancreatic Ductal Adenocarcinoma; MPC: Metastatic Pancreatic Ductal Adenocarcinoma

Table S3 List of the quantified signal and their relative assignment and multiplicity.

Metabolite	Assignment (multiplicity)	matrix	Metabolite	Assignment (multiplicity)	matrix
Formate	8.45 (s)	serum	cholesterol-derivated 1	0.64 (s)	lipid extracts
Unknown signal at 8.12 ppm	8.12 (d)	serum	cholesterol-derivated 2	0.65 (s)	lipid extracts
Unknown signal at 8.07 ppm	8.07 (d)	serum	cholesterol-derivated 3	0.66 (s)	lipid extracts
Phenylalanine	7.42 (m)	serum	cholesterol-derivated 4	0.66 (s)	lipid extracts
Tyrosine	7.19 (m)	serum	cholesterol-derivated 5	0.67 (s)	lipid extracts
Unknown signal at 7.14 ppm	7.14 (m)	serum	free cholesterol	0.68 (s)	lipid extracts
Histidine	7.05 (d)	serum	esterified cholesterol	0.68 (s)	lipid extracts
Glucose	5.23 (d)	serum	cholesterol-derivated 6	0.69 (s)	lipid extracts
Mannose	5.18 (d)	serum	cholesterol-derivated 7	0.70 (s)	lipid extracts
Unknown signal at 5.15 ppm	5.15 (d)	serum	cholesterol-derivated 8	0.70 (s)	lipid extracts
Unknown signal at 5.09 ppm	5.09 (d)	serum	cholesterol-derivated 9	0.71 (s)	lipid extracts
Unknown signal at 5.01 ppm	5.01 (d)	serum	saturated and omega-9 fatty acid	0.87 (t)	lipid extracts
Ascorbate	4.50 (d)	serum	omega-7 fatty acid	0.87 (t)	lipid extracts
Threonine	4.24 (m)	serum	omega-6 fatty acid	0.88 (t)	lipid extracts
Lactate	4.11 (q)	serum	Unknown signal at 0.91 ppm	0.98 (d)	lipid extracts
Creatinine	4.05 (s)	serum	omega-3 fatty acid	0.99 (t)	lipid extracts
Creatine	3.92 (s)	serum	monounsaturated fatty acid	2.01 (q)	lipid extracts
Glycine	3.55 (s)	serum	polyunsaturated fatty acid 1 (except linoleic)	2.80 (t)	lipid extracts
Methanol	3.35 (s)	serum	polyunsaturated fatty acid 2 (except linoleic)	2.84 (d)	lipid extracts
Unknown signal at 2.55 ppm	2.55 (s)	serum	linoleic acid	2.76 (t)	lipid extracts
Citrate	2.53 (d)	serum	phosphatidylcholine	3.20 (s)	lipid extracts
Glutamine	2.45 (m)	serum	sphingomyelin	3.19 (s)	lipid extracts
Pyruvate	2.36 (s)	serum	triglyceride	5.26 (m)	lipid extracts
Glutamate	2.53 (m)	serum	glycerophospholipid	4.45 (q)	lipid extracts
Acetoacetate	2.22 (s)	serum			
Acetate	1.91 (s)	serum			
Alanine	1.47 (d)	serum			
Unknown signal at 1.45 ppm	1.45 (d)	serum			
Unknown signal at 1.43 ppm	1.43 (d)	serum			
3-Hydroxybutyrate	1.19 (d)	serum			
Ethanol	1.17 (t)	serum			
Unknown signal at 1.16 ppm	1.16 (d)	serum			
Unknown signal at 1.14 ppm	1.14 (d)	serum			
Unknown signal at 1.11 ppm	1.11 (d)	serum			
Unknown signal at 1.06 ppm	1.06 (d)	serum			
Valine	1.04 (d)	serum			
Isoleucine	1.00 (d)	serum			
Leucine	0.95 (dd)	serum			
2-Hydroxybutyrate	0.89 (t)	serum			
Protein NH	10.00-6.00	serum			
Unsaturated lipid -CH=CH-	5.50-5.10	serum			
Lipid alpha-CH2	2.25-2.15	serum			
Cholesterol	0.70-0.60	serum			
Lipid =CH-CH2-CH=	2.85-2.65	serum			
Glycerol phospholipid	4.08-4.03	serum			
Phospholipid	3.68-3.62	serum			
Lipid beta-CH2	1.65-1.40	serum			
Lipid CH2	1.40-1.10	serum			
Lipid CH3	1.10-1.08	serum			
GlycB	2.07 (m)	serum			
GlycA	2.03 (m)	serum			

Table S4: Correlation of concentration of metabolites with the stages of Pancreatic Ductal Adenocarcinoma.

Feature	rho	p-value	FDR
Formate	0.22	0.151	0.513
Unknown signal at 8.12 ppm	-0.01	0.941	0.995
Unknown signal at 8.07 ppm	0.3	0.043	0.235
Phenylalanine	0.32	0.030	0.235
Tyrosine	0.01	0.960	0.995
Unknown signal at 7.14 ppm	0.07	0.632	0.849
Histidine	-0.03	0.841	0.933
Glucose	0.13	0.406	0.767
Mannose	0.2	0.181	0.543
Unknown signal at 5.15 ppm	0.18	0.232	0.564
Unknown signal at 5.09 ppm	0.05	0.727	0.905
Unknown signal at 5.01 ppm	0.12	0.439	0.793
Ascorbate	-0.47	0.001	0.021
Threonine	0.16	0.282	0.600
Lactate	0.5	<0.001	0.012
Creatinine	-0.18	0.248	0.574
Creatine	0.08	0.582	0.811
Glycine	0.52	<0.001	0.012
Methanol	-0.19	0.202	0.543
Unknown signal at 2.55 ppm	-0.09	0.542	0.811
Citrate	0	0.997	0.997
Glutamine	-0.11	0.482	0.793
Pyruvate	0.28	0.060	0.278
Glutamate	0.24	0.115	0.450
Acetoacetate	0.1	0.529	0.811
Acetate	0.19	0.200	0.543
Alanine	-0.03	0.822	0.932
Unknown signal at 1.45 ppm	0.32	0.032	0.235
Unknown signal at 1.43 ppm	0.18	0.226	0.564
3-Hydroxybutyrate	0.11	0.467	0.793
Ethanol	0.04	0.804	0.932
Unknown signal at 1.16 ppm	0.15	0.339	0.692
Unknown signal at 1.14 ppm	-0.31	0.035	0.235
Unknown signal at 1.11 ppm	0.31	0.041	0.235
Unknown signal at 1.06 ppm	0.04	0.800	0.932
Valine	-0.2	0.182	0.543
Isoleucine	0.06	0.702	0.895
Leucine	-0.26	0.083	0.354
2-Hydroxybutyrate	0.3	0.046	0.235
Protein NH	-0.42	0.005	0.058
Unsaturated lipid -CH=CH-	-0.08	0.589	0.811
Lipid alpha-CH2	0.17	0.271	0.600
Cholesterol	0.02	0.912	0.989
Lipid =CH-CH2-CH=	-0.13	0.402	0.767
Glycerol phospholipid	0.07	0.658	0.861
Phospholipid	0	0.976	0.995
Lipid beta-CH2	-0.09	0.561	0.811
Lipid CH2	0.05	0.755	0.916
Lipid CH3	-0.1	0.500	0.796
GlycB	0.23	0.129	0.471
GlycA	0.11	0.453	0.793

Rho: spearman's correlation coefficient measures the strength of association between two variables; FDR: false discovery rate

Table S5: Correlation of concentration of lipid extracts with the stages of Pancreatic Ductal Adenocarcinoma.

**Features**	**rho**	**p-value**	**FDR**
cholesterol-derivated 1	0.32	0.047	0.154
cholesterol-derivated 2	0.37	0.018	0.089
cholesterol-derivated 3	0.24	0.133	0.195
cholesterol-derivated 4	0.37	0.019	0.089
cholesterol-derivated 5	-0.29	0.065	0.154
free cholesterol	0.28	0.084	0.159
esterified cholesterol	0.09	0.587	0.587
cholesterol-derivated 6	0.38	0.016	0.089
cholesterol-derivated 7	-0.16	0.313	0.327
cholesterol-derivated 8	0.38	0.014	0.089
cholesterol-derivated 9	-0.33	0.037	0.148
saturated and omega-9 fatty acid	0.3	0.062	0.154
omega-7 fatty acid	0.37	0.017	0.089
omega-6 fatty acid	0.24	0.138	0.195
Unknown signal at 0.91 ppm	-0.29	0.071	0.154
omega-3 fatty acid	0.27	0.086	0.159
monounsaturated fatty acid	0.26	0.110	0.180
polyunsaturated fatty acid 1 (except linoleate)	0.18	0.269	0.293
polyunsaturated fatty acid 2 (except linoleate)	0.23	0.149	0.199
Linoleate	0.21	0.194	0.233
Phosphatidylcholine	0.2	0.206	0.235
Sphingomyelin	0.3	0.065	0.154
Triglyceride	0.21	0.189	0.233
Glycerophospholipid	0.25	0.112	0.180

Rho: spearman's correlation coefficient measures the strength of association between two variables; FDR: false discovery rate

Table S6: Selected metabolites ratios and their catalysing enzymes.

Ratio	Catalysing enzymes
Glucose/Lactate	Phosphofructokinase
Pyruvate/Lactate	Lactate dehydrogenase
Threonine/Glycine	Threonine dehydrogenase<br>2-amino-3-ketobutyrate coenzyme A ligase
Glutamine/Glutamate	Glutamine synthetase
3-hydroxybutyrate/acetoacetate	3-hydroxybutyrate dehydrogenase
Glutamate/Alanine	Alanine aminotransferase

Table S7: Correlation of metabolites ratios with the stages of Pancreatic Ductal Adenocarcinoma.

Feature	rho	p-value	FDR
Glucose/Lactate	-0.19	0.212	0.424
Pyruvate/Lactate	-0.14	0.360	0.599
Threonine/Glycine	-0.09	0.540	0.674
Glutamine/Glutamate	-0.26	0.089	0.222
3-Hydroxybutyrate/Acetoacetate	-0.05	0.767	0.767
Glutamate/Alanine	0.27	0.073	0.222

Rho: spearman's correlation coefficient measures the strength of association between two variables; FDR: false discovery rate

Table S8: Correlation of concentration of lipoproteins with the stages of Pancreatic Ductal Adenocarcinoma.

Feature	rho	p-value	FDR
VLDL cholesterol (nmol/L)	-0.07	0.667	0.754
IDL. cholesterol (nmol/L)	0.32	0.031	0.089
LDL cholesterol (nmol/L)	0.22	0.151	0.230
HDL cholesterol (nmol/L)	-0.38	0.011	0.046
VLDL triglyceride (nmol/L)	-0.03	0.840	0.910
IDL triglyceride (nmol/L)	0.28	0.060	0.139
LDL triglyceride (nmol/L)	0.3	0.043	0.112
HDL triglyceride (nmol/L)	-0.08	0.607	0.717
VLDL particle (nmol/L)	0.01	0.964	0.964
Large VLDL particle(nmol/L)	-0.13	0.404	0.500
Medium VLDL particle (nmol/L)	-0.23	0.121	0.211
Small VLDL particle(nmol/L)	0.02	0.916	0.953
LDL particle (nmol/L)	0.26	0.080	0.161
Large LDL particle (nmol/L)	0.21	0.174	0.251
Medium LDL particle (nmol/L)	0.28	0.064	0.139
Small LDL particle (nmol/L)	0.15	0.333	0.450
HDL particle (mol/L)	-0.39	0.008	0.046
Large HDL particle (mol/L)	-0.35	0.019	0.072
Medium HDL particle (mol/L)	-0.39	0.008	0.046
Small HDL particle (mol/L)	-0.39	0.009	0.046
VLDL size (nm)	-0.22	0.139	0.225
LDL size (nm)	0.14	0.346	0.450
HDL size (nm)	0.34	0.024	0.077
Non-HDL particle (nmol/L)	0.26	0.087	0.162
Total particle / HDL particle (nmol/L)	0.39	0.008	0.046
LDL particle / HDL particle (nmol/L)	0.39	0.008	0.046

Rho: spearman's correlation coefficient measures the strength of association between two variables; FDR: false discovery rate

Table S9: Correlation of metabolites with survival time.

Features	p-value	FDR
Formate	0.657	0.964
Unknown signal at 8.12 ppm	0.169	0.792
Unknown signal at 8.07 ppm	0.237	0.810
Phenylalanine	0.615	0.964
Tyrosine	0.895	0.964
Unknown signal at 7.14 ppm	0.865	0.964
Histidine	0.707	0.964
Glucose	0.919	0.964
Mannose	0.186	0.792
Unknown signal at 5.15 ppm	0.549	0.964
Unknown signal at 5.09 ppm	0.900	0.964
Unknown signal at 5.01 ppm	0.312	0.842
Ascorbate	0.379	0.919
Threonine	0.347	0.886
Lactate	0.852	0.964
Creatinine	0.929	0.964
Creatine	0.692	0.964
Glycine	0.236	0.810
Methanol	0.431	0.964
Unknown signal at 2.55 ppm	0.527	0.964
Citrate	0.083	0.760
Glutamine	0.500	0.964
Pyruvate	0.826	0.964
Glutamate	0.591	0.964
Acetoacetate	0.089	0.760
Acetate	0.837	0.964
Alanine	0.945	0.964
Unknown signal at 1.45 ppm	0.036	0.606
Unknown signal at 1.43 ppm	0.160	0.792
3-Hydroxybutyrate	0.015	0.370
Ethanol	0.002	0.126
Unknown signal at 1.16 ppm	0.152	0.792
Unknown signal at 1.14 ppm	0.643	0.964
Unknown signal at 1.11 ppm	0.974	0.974
Unknown signal at 1.06 ppm	0.634	0.964
Valine	0.811	0.964
Isoleucine	0.512	0.964
Leucine	0.699	0.964
2-Hydroxybutyrate	0.181	0.792
Protein NH	0.791	0.964
Unsaturated lipid -CH=CH-	0.451	0.964
Lipid alpha-CH2	0.908	0.964
Cholesterol	0.059	0.752
Lipid =CH-CH2-CH=	0.270	0.810
Glycerol phospholipid	0.570	0.964
Phospholipid	0.159	0.792
Lipid beta-CH2	0.560	0.964
Lipid CH2	0.763	0.964
Lipid CH3	0.314	0.842
GlycB	0.269	0.810
GlycA	0.257	0.810

FDR: false discovery rate

Table S10: Correlation of the lipid extracts with survival time.

Feature	p-value	FDR
cholesterol-derivated 1	0.646	0.948
cholesterol-derivated 2	0.664	0.948
cholesterol-derivated 3	0.745	0.948
cholesterol-derivated 4	0.783	0.948
cholesterol-derivated 5	0.854	0.948
free cholesterol	0.722	0.948
esterified cholesterol	0.425	0.948
cholesterol-derivated 6	0.245	0.948
cholesterol-derivated 7	0.583	0.948
cholesterol-derivated 8	0.785	0.948
cholesterol-derivated 9	0.575	0.948
saturated and omega-9 fatty acid	0.710	0.948
omega-7 fatty acid	0.995	0.995
omega-6 fatty acid	0.486	0.948
Unknown signal at	0.508	0.948
omega-3 fatty acid	0.699	0.948
monounsaturated fatty acid	0.882	0.948
polyunsaturated fatty acid 1 (except linoleic)	0.588	0.948
polyunsaturated fatty acid 2 (except linoleic)	0.619	0.948
linoleic acid	0.908	0.948
phosphatidylcholine	0.697	0.948
sphingomyelin	0.603	0.948
triglyceride	0.682	0.948
glycerophospholipid	0.817	0.948

FDR: false discovery rate

Table S11: Correlation of metabolite concentration ratios with survival time.

Features	p-value	FDR
Glucose/Lactate	0.741	0.873
Pyruvate/Lactate	0.873	0.873
Threonine/Glycine	0.721	0.873
Glutamine/Glutamate	0.473	0.788
3-Hydroxybutyrate/Acetoacetate	0.241	0.783
Glutamate/Alanine	0.313	0.783

FDR: false discovery rate

Table S12: Correlation of lipoproteins with survival time.

Features	p-value	FDR
VLDL cholesterol (nmol/L)	0.705	0.971
IDL. cholesterol (nmol/L)	0.699	0.971
LDL cholesterol (nmol/L)	0.174	0.971
HDL cholesterol (nmol/L)	0.666	0.971
VLDL triglyceride (nmol/L)	0.824	0.971
IDL triglyceride (nmol/L)	0.718	0.971
LDL triglyceride (nmol/L)	0.623	0.971
HDL triglyceride (nmol/L)	0.467	0.971
VLDL particle (nmol/L)	0.996	0.996
Large VLDL particle(nmol/L)	0.771	0.971
Medium VLDL particle (nmol/L)	0.581	0.971
Small VLDL particle(nmol/L)	0.935	0.973
LDL particle (nmol/L)	0.250	0.971
Large LDL particle (nmol/L)	0.324	0.971
Medium LDL particle (nmol/L)	0.379	0.971
Small LDL particle (nmol/L)	0.136	0.971
HDL particle (mol/L)	0.815	0.971
Large HDL particle (mol/L)	0.738	0.971
Medium HDL particle (mol/L)	0.884	0.971
Small HDL particle (mol/L)	0.627	0.971
VLDL size (nm)	0.776	0.971
LDL size (nm)	0.871	0.971
HDL size (nm)	0.404	0.971
Non-HDL particle (nmol/L)	0.271	0.971
Total particle / HDL particle (nmol/L)	0.866	0.971
LDL particle / HDL particle (nmol/L)	0.896	0.971

FDR: false discovery rate

Table S13: Comparison of the Full Blood Count of N versus AB Clusters.

Features	N, median [IQR]	AB, median [IQR]	log change	p-value	FDR
Haematocrit	0.354 [0.312 0.392]	0.28 [0.261 0.306]	-0.32	0.003	0.015
White cell count (/L^9)	7.315 [5.772 10.12]	10.8 [8.27 15.04]	0.73	0.011	0.040
Haemaglobin (g/DL)	11.2 [10.2 12.95]	9.15 [8.725 9.85]	-0.29	0.002	0.015
Platelet count (/L^9)	352.5 [250.75 450.5]	410 [351.5 460.75]	0.25	0.137	0.250
Prothrombin time (Sec)	13.3 [12.4 14.8]	14.2 [12.8 14.475]	0.05	0.719	0.798
INR	1.08 [0.99 1.19]	1.115 [1.032 1.168]	0.06	0.639	0.798
MCV (fL)	92.3 [87.8 96.5]	89.35 [87.625 95.65]	0	0.826	0.826
MCH (pg)	30.2 [28.1 31.4]	29.8 [28.825 32.1]	0.03	0.725	0.798
MCHC (g/dl)	32.7 [31.3 33.4]	33.5 [32.2 34.15]	0.03	0.131	0.250
MPV (fL)	10.5 [9.475 10.8]	10.75 [9.825 11.2]	0.05	0.172	0.271
Red cell count (/L^12)	3.77 [3.42 4.54]	2.985 [2.75 3.473]	-0.12	0.020	0.055

IQR: Interquartile range; FDR: false discovery rate

Table S14: Comparison of the Blood chemistry of N versus AB components.

Feature	N, median [IQR]	AB, median [IQR]	log change	p-value	FDR
Sodium (mmol/L)	138 [135 140]	136.5 [130.5 138.5]	-0.04	0.063	0.260
Potassium (mmol/L)	3.8 [3.4 4.5]	3.65 [3.35 3.875]	-0.08	0.403	0.448
Chloride (mmol/L)	97 [93 99]	93 [91.25 96]	-0.04	0.078	0.260
Bicarbonate (mmol/L)	21 [19 24]	18.5 [17 24]	-0.14	0.186	0.321
Anion gap (mmol/L)	23 [20 27]	27.5 [21.25 30.75]	0.07	0.395	0.448
Calcium(mmol/L)	2.2 [2.13 2.32]	2.14 [1.997 2.223]	-0.14	0.107	0.267
Magnesium (mmol/L)	0.84 [0.79 0.92]	0.72 [0.673 0.753]	-0.29	0.019	0.190
Inorganic Phosphate (mmol/L)	1.13 [1.03 1.26]	1.02 [0.938 1.198]	-0.16	0.192	0.321
Urea (mmol/L)	4.3 [2.9 5.6]	3.25 [2 8.175]	0.17	0.747	0.747
Creatinine (µmol/L)	75 [63 97]	62.5 [52.75 88.75]	-0.22	0.266	0.380

IQR: Interquartile range; FDR: false discovery rate

Table S15: Comparison of the Liver function tests of N versus AB components.

Feature	N, median [IQR]	AB, median [IQR]	log change	p-val	FDR
Total Protein (g/L)	68 [59 76]	58 [53 64]	-0.23	0.018	0.029
Albumin (g/L)	36 [28 40]	27.5 [26.25 29.75]	-0.34	0.002	0.012
Total Bilirubin (µmol/L)	43 [8 100]	174.5 [143.25 266]	1.32	0.003	0.012
Conjugated Bilirubin (µmol/L)	31 [3 97]	146 [126.75 241.5]	1.34	0.006	0.016
Alanine transaminase (U/L)	28 [18 71]	81.5 [23.25 113.25]	0.2	0.371	0.425
Aspartate transaminase (U/L)	42 [24 92]	115.5 [83 148.5]	0.82	0.009	0.018
Alkaline phosphatase (U/L)	288 [103 683]	696 [330.25 1378.25]	0.62	0.044	0.059
Gamma glutamyl transferase (U/L)	307 [77 1021]	394 [208 926.25]	-0.04	0.650	0.650

IQR: Interquartile range; FDR: false discovery rate

Table S16: Comparison of the metabolite concentration of N versus AB clusters.

Features	N (mM), median [IQR]	AB (mM), median [IQR]	log change	p-value	FDR
Formate	0.016 [0.011 0.019]	0.019 [0.016 0.022]	0.24	0.067	0.225
Unknown signal at 8.12 ppm	0 [0 0.007]	0 [0 0.023]	1.18	0.304	0.550
Unknown signal at 8.07 ppm	0 [0 0.005]	0 [0 0.01]	1	0.193	0.411
Phenylalanine	0.104 [0.069 0.128]	0.113 [0.102 0.143]	0.14	0.080	0.225
Tyrosine	0.046 [0.036 0.052]	0.04 [0.035 0.054]	-0.44	0.585	0.787
Unknown signal at 7.14 ppm	0.026 [0 0.079]	0.034 [0 0.052]	-0.78	0.820	0.889
Histidine	0.064 [0.055 0.082]	0.074 [0.064 0.078]	-0.01	0.313	0.550
Glucose	3.532 [3.029 4.205]	3.549 [2.546 4.962]	0.13	0.952	0.980
Mannose	0.037 [0.028 0.051]	0.078 [0.037 0.115]	1.06	0.005	0.029
Unknown signal at 5.15 ppm	0 [0 0]	0 [0 0]	0.23	0.607	0.787
Unknown signal at 5.09 ppm	0 [0 0.043]	0 [0 0.032]	-0.67	0.696	0.845
Unknown signal at 5.01 ppm	0 [0 0]	0 [0 0]	0.99	0.151	0.340
Ascorbate	0.01 [0 0.019]	0 [0 0.014]	-0.6	0.397	0.621
Threonine	0.079 [0.06 0.091]	0 [0 0.043]	-1.47	0.002	0.013
Lactate	2.253 [2.013 3.114]	2.927 [2.62 3.179]	-0.01	0.153	0.340
Creatinine	0.057 [0.044 0.073]	0.053 [0.031 0.076]	-0.11	0.520	0.781
Creatine	0.012 [0.006 0.035]	0.002 [0 0.028]	-0.56	0.089	0.227
Glycine	0.318 [0.242 0.354]	0.311 [0.273 0.353]	-0.12	0.952	0.980
Methanol	0.027 [0.018 0.036]	0.021 [0.013 0.023]	-0.55	0.054	0.219
Unknown signal at 2.55 ppm	0 [0 0.013]	0 [0 0.013]	-3.27	0.589	0.787
Citrate	0.081 [0 0.103]	0.09 [0 0.123]	-0.11	0.100	0.100
Glutamine	0.4 [0.294 0.424]	0.268 [0.197 0.294]	-0.57	<0.001	0.007
Pyruvate	0.058 [0.048 0.072]	0.061 [0.047 0.091]	0.08	0.762	0.883
Glutamate	0.19 [0.129 0.272]	0.202 [0.155 0.285]	0.09	0.402	0.621
Acetoacetate	0.019 [0.016 0.041]	0.025 [0.017 0.089]	0.09	0.301	0.550
Acetate	0.024 [0.02 0.033]	0.032 [0.026 0.039]	0.09	0.120	0.292
Alanine	0.395 [0.293 0.481]	0.341 [0.273 0.35]	-0.56	0.051	0.219
Unknown signal at 1.45 ppm	0.031 [0.024 0.039]	0.02 [0 0.049]	-0.18	0961	0.980
Unknown signal at 1.43 ppm	0 [0 0]	0 [0 0.405]	1.85	0.005	0.029
3-Hydroxybutyrate	0.046 [0.028 0.1]	0.05 [0.037 0.451]	0.73	0.580	0.787
Ethanol	0.022 [0 0.083]	0 [0 0.084]	-0.81	0.389	0.621
Unknown signal at 1.16 ppm	0.112 [0 0.297]	0.168 [0.026 0.337]	0.08	0.610	0.787
Unknown signal at 1.14 ppm	0.019 [0 0.043]	0.016 [0 0.029]	-0.81	0.729	0.865
Unknown signal at 1.11 ppm	0.053 [0.044 0.061]	0.061 [0.041 0.073]	0.01	0.633	0.787
Unknown signal at 1.06 ppm	0.029 [0.021 0.038]	0.044 [0.026 0.067]	0.24	0.057	0.219
Valine	0.163 [0.127 0.183]	0.139 [0.105 0.161]	-0.35	0.084	0.225
Isoleucine	0.041 [0.033 0.053]	0.046 [0.042 0.054]	0.06	0.391	0.621
Leucine	0.068 [0.055 0.075]	0.05 [0.042 0.067]	-0.59	0.021	0.108
2-Hydroxybutyrate	0 [0 0.021]	0.009 [0 0.048]	1.07	0.312	0.550
Protein NH	144.358 [121.287 154.93]	105.798 [104.188 112.176]	-0.36	<0.001	<0.001
Unsaturated lipid -CH=CH-	11.141 [8.332 14.097]	14.379 [9.344 19.988]	0.36	0.080	0.225
Lipid alpha-CH2	1.863 [1.252 2.379]	6.257 [4.119 8.056]	1.7	<0.001	<0.001
Cholesterol	0.825 [0.644 1.064]	0.516 [0.27 0.934]	-0.37	0.060	0.219
Lipid =CH-CH2-CH=	4.954 [3.999 6.138]	5.02 [3.569 7.556]	0.17	0.818	0.889
Glycerol phospholipid	0.487 [0.243 0.77]	1.663 [1.412 2.797]	1.94	<0.001	<0.001
Phospholipid	3.699 [3.478 4.142]	3.128 [2.95 3.39]	-0.24	<0.001	0.005
Lipid beta-CH2	7.403 [5.083 8.55]	10.095 [6.281 12.356]	0.35	0.084	0.225
Lipid CH2	62.251 [55.441 75.596]	111.18 [73.472 140.862]	0.71	<0.001	0.001
Lipid CH3	26.385 [21.436 29.909]	27.943 [23.387 34.927]	0.14	0.279	0.550
GlycB	0.406 [0.322 0.471]	0.407 [0.366 0.436]	-0.04	0.799	0.889
GlycA	1.996 [1.535 2.186]	1.833 [1.701 2.005]	-0.03	0.619	0.787

IQR: Interquartile range; FDR: false discovery rate

Table S17: Comparison of the lipid extracts concentration of N versus AB clusters.

Feature	N, median [IQR]	AB, median [IQR]	log change	p-value	FDR
cholesterol-derivated 1	0.661 [0.571 0.791]	1.46 [1.158 1.675]	0.88	<0.001	<0.001
cholesterol-derivated 2	0.613 [0.537 0.75]	1.484 [1.15 1.745]	0.97	<0.001	<0.001
cholesterol-derivated 3	0.805 [0.694 0.893]	1.273 [1.118 1.327]	0.48	<0.001	<0.001
cholesterol-derivated 4	0.639 [0.569 0.778]	1.424 [1.185 1.641]	0.85	<0.001	<0.001
cholesterol-derivated 5	1.299 [0.909 1.667]	0.271 [0.241 0.385]	-2.14	<0.001	<0.001
free cholesterol	0.193 [0.055 0.271]	2.095 [0.96 3.516]	2.52	<0.001	<0.001
esterified cholesterol	0.93 [0.572 1.271]	1.073 [0.343 1.437]	-0.03	0.977	0.977
cholesterol-derivated 6	0.608 [0.526 0.828]	1.54 [0.835 2.05]	0.99	0.002	0.002
cholesterol-derivated 7	1.069 [0.907 1.394]	0.713 [0.604 0.784]	-0.67	<0.001	<0.001
cholesterol-derivated 8	0.607 [0.547 0.822]	1.468 [1.162 1.69]	0.9	<0.001	<0.001
cholesterol-derivated 9	1.127 [0.747 1.75]	0.333 [0.31 0.401]	-1.87	<0.001	<0.001
saturated and omega-9 fatty acid	0.667 [0.538 0.833]	1.443 [1.223 1.999]	1.07	<0.001	<0.001
omega-7 fatty acid	0.909 [0.78 1.043]	1.126 [0.982 1.221]	0.36	0.002	0.002
omega-6 fatty acid	0.583 [0.367 1.078]	1.448 [1.256 1.851]	1.07	<0.001	<0.001
Unknown signal at 0.91 ppm	0 [0 0]	0 [0 0]	-	0.230	0.240
omega-3 fatty acid	0.626 [0.397 0.798]	1.748 [1.159 1.915]	1.14	<0.001	<0.001
monounsaturated fatty acid	0.38 [0.193 0.656]	1.898 [1.386 2.441]	2.06	<0.001	<0.001
Polyunsaturated fatty acid 1	0.552 [0.342 0.832]	1.63 [1.211 2.031]	1.46	<0.001	<0.001
Polyunsaturated fatty acid 2	0.536 [0.334 0.812]	1.736 [1.327 2.057]	1.47	<0.001	<0.001
linoleic acid	0.476 [0.228 0.756]	1.582 [1.234 2.454]	1.53	<0.001	<0.001
phosphatidylcholine	0.533 [0.419 0.669]	1.624 [1.108 2.288]	1.52	<0.001	<0.001
sphingomyelin	0.594 [0.499 0.833]	1.205 [0.928 1.719]	0.97	<0.001	<0.001
triglyceride	0.59 [0.474 0.838]	1.643 [1.413 2.011]	1.41	<0.001	<0.001
glycerophospholipid	0.494 [0.393 0.614]	1.704 [1.26 2.384]	1.65	<0.001	<0.001

IQR: Interquartile range; FDR: false discovery rate

Table S18: Comparison of the metabolite concentration ratios of N versus AB clusters.

Feature	N, median [IQR]	AB, median [IQR]	log change	p-value	FDR
Glucose/Pyruvate	1.483 [1.254 1.769]	1.201 [0.975 1.774]	0.10	0.459	0.689
Pyruvate/Lactate	0.023 [0.019 0.031]	0.027 [0.017 0.029]	-0.05	0.799	0.912
Threonine/Glycine	0.248 [0.207 0.31]	0 [0 0.152]	-1.51	0.004	0.019
Glutamine/Glutamate	1.966 [1.23 3.538]	1.378 [0.704 2.113]	-0.80	0.029	0.048
3-Hydroxybutyrate/Acetoacetate	2.566 [1.258 3.729]	2.415 [0.865 4.247]	0.11	0.912	0.912
Glutamate/Alanine	0.52 [0.358 0.606]	0.634 [0.516 0.976]	0.59	0.013	0.031

IQR: Interquartile range; FDR: false discovery rate

Table S19: Comparison of the lipoprotein profile for N versus AB clusters.

Feature	N, median [IQR]	AB, median [IQR]	log change	p-value	FDR
VLDL cholesterol (nmol/L)	17.81 [11.205 24.26]	24.08 [16.92 34.872]	0.58	0.025	0.030
IDL. cholesterol (nmol/L)	17.31 [10.345 21.475]	73.96 [52.33 82.738]	1.91	<0.001	<0.001
LDL cholesterol (nmol/L)	108.36 [87.245 130.24]	146.94 [119.998 165.042]	0.38	0.008	0.010
HDL cholesterol (nmol/L)	36.31 [28.005 50.43]	1 [1 1]	-4.99	<0.001	<0.001
VLDL triglyceride (nmol/L)	73.84 [47.195 92.89]	108.86 [82.815 141.52]	0.77	<0.001	0.001
IDL triglyceride (nmol/L)	15.4 [11.215 18.165]	50.75 [35.712 57.97]	1.55	<0.001	<0.001
LDL triglyceride (nmol/L)	18.96 [11.86 30.375]	72.13 [48.785 85.205]	1.59	<0.001	<0.001
HDL triglyceride (nmol/L)	17.76 [15.12 22.85]	24.345 [13.485 30.7]	0.25	0.459	0.459
VLDL particle (nmol/L)	50.87 [33.49 65.585]	79.76 [56.802 104.332]	0.75	<0.001	0.001
Large VLDL particle(nmol/L)	1.38 [0.875 1.61]	1.445 [1.375 1.7]	0.39	0.095	0.103
Medium VLDL particle (nmol/L)	5.53 [4.03 7.4]	5.465 [5.07 7.022]	0.31	0.440	0.458
Small VLDL particle(nmol/L)	43.15 [29.4 56.13]	74.645 [50.73 96.23]	0.8	<0.001	<0.001
LDL particle (nmol/L)	1113.73 [848.825 1331.92]	1745.03 [1458.052 2184.075]	0.66	<0.001	<0.001
Large LDL particle (nmol/L)	180.92 [131.475 215.42]	268.655 [194.603 301.295]	0.45	0.002	<0.001
Medium LDL particle (nmol/L)	349.05 [194.215 521.08]	868.56 [666.858 1136.122]	1.12	<0.001	<0.001
Small LDL particle (nmol/L)	577.17 [480.125 661.975]	634.715 [559.785 805.162]	0.29	0.084	0.095
HDL particle (mol/L)	18.54 [14.905 25.96]	4.855 [2.812 5.335]	-2.13	<0.001	<0.001
Large HDL particle (mol/L)	0.28 [0.245 0.305]	0.175 [0.105 0.223]	-0.78	<0.001	<0.001
Medium HDL particle (mol/L)	10.55 [9.295 11.395]	5.735 [3.708 6.16]	-1	<0.001	<0.001
Small HDL particle (mol/L)	8.87 [3.175 15.98]	0 [0 0]	-5.7	<0.001	<0.001
VLDL size (nm)	42.2 [42.175 42.22]	42.16 [42.13 42.18]	0	0.001	0.002
LDL size (nm)	21.27 [21.135 21.46]	21.595 [21.505 21.655]	0.02	<0.001	<0.001
HDL size (nm)	8.5 [8.34 8.9]	9.55 [9.47 9.645]	0.15	<0.001	<0.001
Non-HDL particle (nmol/L)	1130.02 [847.865 1358.64]	1838.72 [1510.072 2274.648]	0.68	<0.001	<0.001
Total particle / HDL particle (nmol/L)	48.14 [35.675 98.015]	437.875 [354.01 539.432]	2.64	<0.001	<0.001
LDL particle / HDL particle (nmol/L)	43.99 [34.045 95.405]	414.975 [333.365 517.24]	2.63	<0.001	<0.001

IQR: Interquartile range; FDR: false discovery rate

Table S20: Correlation of metabolites with GlycA, GlycB, CRP, and Albumin intensity value.

	GlycA			GlycB			CRP			Albumin		
**Feature**	**rho**	**p-value**	**FDR**	**rho**	**p-value**	**FDR**	**rho**	**p-value**	**FDR**	**rho**	**p-value**	**FDR**
Formate	0.2	0.186	0.389	0.27	0.072	0.334	0.08	0.656	0.910	-0.27	0.101	0.477
Unknown signal at 8.12 ppm	-0.27	0.068	0.233	-0.23	0.122	0.367	-0.08	0.636	0.910	-0.14	0.387	0.658
Unknown signal at 8.07 ppm	-0.26	0.079	0.233	-0.16	0.304	0.583	0.03	0.847	0.910	-0.19	0.237	0.526
Phenylalanine	0.2	0.190	0.389	0.29	0.055	0.280	0.29	0.079	0.808	-0.21	0.197	0.523
Tyrosine	-0.33	0.027	0.175	-0.26	0.079	0.335	0.06	0.727	0.910	0.06	0.702	0.904
Unknown signal at 7.14 ppm	0.34	0.021	0.157	0.26	0.090	0.346	0.08	0.622	0.910	-0.26	0.115	0.477
Histidine	-0.56	<0.001	0.001	-0.49	<0.001	0.008	-0.16	0.358	0.829	0.07	0.662	0.904
Glucose	0.27	0.727	0.233	0.36	0.015	0.095	-0.22	0.184	0.829	0.05	0.740	0.904
Mannose	0.34	0.021	0.157	0.36	0.115	0.095	0.04	0.832	0.910	-0.21	0.192	0.523
Unknown signal at 5.15 ppm	-0.25	0.096	0.233	-0.14	0.358	0.593	-0.06	0.738	0.910	-0.18	0.269	0.553
Unknown signal at 5.09 ppm	0.26	0.086	0.233	0.18	0.233	0.583	-0.01	0.950	0.969	-0.29	0.076	0.477
Unknown signal at 5.01 ppm	-0.28	0.061	0.233	-0.16	0.286	0.583	0.07	0.692	0.910	-0.24	0.140	0.477
Ascorbate	-0.25	0.095	0.233	-0.25	0.099	0.346	-0.05	0.749	0.910	-0.04	0.806	0.909
Threonine	0.04	0.807	0.895	0.02	0.878	0.904	-0.16	0.344	0.829	0.3	0.064	0.477
Lactate	0.32	0.031	0.176	0.38	0.009	0.082	0.4	0.014	0.559	-0.2	0.218	0.523
Creatinine	-0.1	0.506	0.842	-0.06	0.693	0.803	-0.06	0.711	0.910	-0.04	0.820	0.909
Creatine	-0.06	0.675	0.842	-0.03	0.862	0.904	0.2	0.228	0.829	0	0.993	0.993
Glycine	0.07	0.643	0.842	0.13	0.397	0.610	0.16	0.356	0.829	-0.22	0.180	0.523
Methanol	-0.03	0.848	0.920	-0.18	0.237	0.583	0.04	0.830	0.910	0.35	0.028	0.283
Unknown signal at 2.55 ppm	0.08	0.619	0.842	-0.05	0.768	0.851	-0.16	0.341	0.829	0.22	0.169	0.523
Citrate	-0.18	0.237	0.465	-0.06	0.710	0.805	-0.08	0.642	0.910	-0.09	0.599	0.872
Glutamine	-0.06	0.694	0.842	-0.16	0.300	0.583	-0.25	0.137	0.829	0.05	0.773	0.909
Pyruvate	0.15	0.340	0.598	0.24	0.109	0.346	0.19	0.270	0.829	-0.24	0.135	0.477
Glutamate	0.02	0.879	0.934	0.07	0.645	0.783	0.09	0.595	0.910	-0.06	0.733	0.904
Acetoacetate	-0.3	0.049	0.233	-0.16	0.283	0.583	-0.03	0.857	0.910	-0.12	0.455	0.737
Acetate	-0.17	0.277	0.523	-0.08	0.596	0.760	-0.03	0.857	0.910	-0.16	0.318	0.588
Alanine	-0.1	0.531	0.842	-0.12	0.413	0.610	-0.07	0.695	0.910	0.25	0.124	0.477
Unknown signal at 1.45 ppm	0.6	<0.001	<0.001	0.65	<0.001	<0.001	0.36	0.028	0.559	-0.07	0.657	0.904
Unknown signal at 1.43 ppm	-0.29	0.055	0.233	-0.24	0.108	0.346	0.04	0.814	0.910	-0.28	0.087	0.477
3-Hydroxybutyrate	-0.06	0.677	0.842	0	0.977	0.977	0.12	0.475	0.910	-0.06	0.708	0.904
Ethanol	-0.05	0.726	0.842	-0.06	0.673	0.798	0	0.998	0.998	-0.11	0.493	0.761
Unknown signal at 1.16 ppm	0.06	0.713	0.842	0.12	0.430	0.610	-0.16	0.347	0.829	-0.16	0.320	0.588
Unknown signal at 1.14 ppm	0.22	0.149	0.330	0.15	0.338	0.593	0.17	0.321	0.829	0.12	0.462	0.737
Unknown signal at 1.11 ppm	0	0.999	0.999	0.11	0.490	0.657	-0.07	0.697	0.910	0	0.993	0.993
Unknown signal at 1.06 ppm	-0.26	0.090	0.233	-0.17	0.257	0.583	0.12	0.480	0.910	-0.16	0.323	0.588
Valine	-0.08	0.611	0.842	-0.12	0.443	0.611	-0.07	0.682	0.910	0.2	0.226	0.523
Isoleucine	0.25	0.103	0.238	0.15	0.325	0.591	-0.02	0.920	0.957	-0.11	0.510	0.766
Leucine	0.09	0.536	0.842	0.09	0.572	0.748	0.09	0.585	0.910	0.26	0.116	0.477
2-Hydroxybutyrate	-0.28	0.065	0.233	-0.14	0.361	0.593	0.12	0.465	0.910	-0.03	0.880	0.954
Protein NH	0.08	0.610	0.842	-0.03	0.831	0.902	-0.17	0.317	0.829	0.7	<0.001	<0.001
Unsaturated lipid -CH=CH-	-0.09	0.567	0.842	-0.16	0.286	0.583	0.23	0.174	0.829	0.04	0.786	0.909
Lipid alpha-CH2	-0.09	0.567	0.842	-0.13	0.397	0.610	0.33	0.043	0.559	-0.43	0.006	0.157
Cholesterol	0.01	0.926	0.964	-0.02	0.886	0.904	-0.04	0.825	0.910	0.27	0.099	0.477
Lipid =CH-CH2-CH=	0	0.986	0.999	-0.08	0.621	0.773	0.2	0.230	0.829	0.18	0.271	0.553
Glycorol phospholipid	-0.16	0.306	0.557	-0.21	0.172	0.488	0.25	0.143	0.829	-0.37	0.019	0.243
Phospholipid	0.42	0.004	0.041	0.41	0.005	0.055	0.06	0.703	0.910	0.4	0.012	0.198
Lipid beta-CH2	-0.27	0.073	0.233	-0.32	0.031	0.178	0.13	0.458	0.910	-0.02	0.917	0.974
Lipid CH2	-0.07	0.647	0.842	-0.15	0.309	0.583	0.33	0.043	0.559	-0.2	0.213	0.523
Lipid CH3	-0.04	0.801	0.895	-0.12	0.424	0.610	0.19	0.262	0.829	0.16	0.340	0.598
GlycB	0.9	<0.001	<0.001	1	0	0	0.22	0.195	0.829	0.01	0.969	0.993
GlycA	1	0	0	0.9	<0.001	<0.001	0.26	0.115	0.829	0.05	0.744	0.904

Rho: spearman's correlation coefficient measures the strength of association between two variables; FDR: false discovery rate

Table S21: Correlation of lipid extracts with GlycA, GlycB, CRP, and Albumin intensity value.

	GlycA			GlycB			CRP			Albumin		
**Feature**	**rho**	**p-value**	**FDR**	**rho**	**p-value**	**FDR**	**rho**	**p-value**	**FDR**	**rho**	**p-value**	**FDR**
cholesterol-derivated 1	-0.17	0.296	0.666	-0.11	0.502	0.788	0.35	0.045	0.123	-0.54	<0.001	0.006
cholesterol-derivated 2	-0.16	0.328	0.666	-0.1	0.525	0.788	0.37	0.035	0.123	-0.53	0.001	0.006
cholesterol-derivated 3	-0.22	0.172	0.666	-0.17	0.282	0.788	0.41	0.019	0.123	-0.5	0.002	0.007
cholesterol-derivated 4	-0.17	0.299	0.666	-0.12	0.458	0.788	0.37	0.035	0.123	-0.54	<0.001	0.006
cholesterol-derivated 5	0.11	0.511	0.756	0.05	0.775	0.829	-0.35	0.044	0.123	0.51	0.002	0.006
free cholesterol	-0.18	0.273	0.666	-0.13	0.440	0.788	0.3	0.094	0.174	-0.39	0.020	0.044
esterified cholesterol	-0.04	0.815	0.857	-0.13	0.414	0.788	-0.09	0.632	0.632	0.34	0.044	0.082
cholesterol-derivated 6	-0.14	0.397	0.666	-0.04	0.798	0.829	0.26	0.146	2.00	-0.56	<0.001	0.060
cholesterol-derivated 7	-0.06	0.701	0.857	-0.04	0.829	0.829	-0.35	0.046	0.123	0.27	0.117	0.165
cholesterol-derivated 8	-0.16	0.338	0.666	-0.11	0.490	0.788	0.37	0.035	0.123	-0.51	0.002	0.006
cholesterol-derivated 9	0.1	0.535	0.756	0.1	0.545	0.788	-0.39	0.027	0.123	0.51	0.002	0.006
saturated and omega-9 fatty acid	-0.23	0.156	0.666	-0.14	0.372	0.788	0.32	0.071	0.172	-0.44	0.009	0.022
omega-7 fatty acid	-0.03	0.857	0.857	-0.05	0.736	0.829	0.18	0.321	0.335	-0.47	0.004	0.011
omega-6 fatty acid	-0.07	0.648	0.857	-0.1	0.558	0.788	0.23	0.196	0.235	-0.19	0.285	0.285
Unknown signal at 0.91 ppm	-0.03	0.837	0.857	-0.11	0.488	0.788	0.19	0.288	0.314	-0.24	0.163	0.218
omega-3 fatty acid	-0.16	0.323	0.666	-0.09	0.602	0.803	0.2	0.273	0.312	-0.29	0.095	0.142
monounsaturated fatty acid	-0.13	0.417	0.666	-0.1	0.539	0.788	0.3	0.093	0.174	-0.36	0.033	0.065
Polyunsaturated fatty acid 1	-0.14	0.376	0.666	-0.13	0.425	0.788	0.28	0.110	0.188	-0.19	0.284	0.285
Polyunsaturated fatty acid 2	-0.19	0.245	0.666	-0.16	0.326	0.788	0.26	0.150	2.00	-0.21	0.233	0.266
linoleic acid	-0.17	0.282	0.666	-0.14	0.390	0.788	0.26	0.147	2.00	-0.22	0.200	0.240
phosphatidylcholine	-0.19	0.237	0.666	-0.16	0.316	0.788	0.27	0.130	2.00	-0.32	0.063	0.100
sphingomyelin	-0.05	0.753	0.857	-0.04	0.814	0.829	0.3	0.091	0.174	-0.19	0.274	0.285
triglyceride	-0.04	0.825	0.857	-0.06	0.724	0.829	0.35	0.043	0.123	-0.23	0.176	0.222
glycerophospholipid	-0.15	0.347	0.666	-0.11	0.481	0.788	0.23	0.196	0.235	-0.33	0.050	0.086

Rho: spearman's correlation coefficient measures the strength of association between two variables; FDR: false discovery rate

Table S22: Correlation of metabolite concentration ratios with GlycA, GlycB, CRP, and Albumin intensity value.

	GlycA			GlycB			CRP			Albumin		
**Feature**	**rho**	**p-value**	**FDR**	**rho**	**p-value**	**FDR**	**rho**	**p-value**	**FDR**	**rho**	**p-value**	**FDR**
Glucose/Lactate	-0.03	0.867	0.960	0.01	0.970	0.970	-0.44	0.006	0.004	0.19	0.256	0.683
Pyruvate/Lactate	-0.08	0.622	0.778	-0.07	0.631	0.631	-0.09	0.585	0.585	-0.08	0.644	0.683
Threonine/Glycine	-0.01	0.960	0.960	-0.08	0.584	0.631	-0.36	0.029	0.143	0.48	0.002	0.011
Glutamine/Glutamate	-0.09	0.568	0.778	-0.13	0.381	0.631	-0.18	0.286	0.408	0.07	0.683	0.683
3Hydroxybutyrate/Acetoacetate	0.12	0.431	0.778	0.12	0.450	0.631	0.24	0.160	0.399	-0.07	0.661	0.683
Glutamate/Alanine	0.11	0.473	0.778	0.12	0.417	0.631	0.17	0.326	0.408	-0.13	0.444	0.683

Rho: spearman's correlation coefficient measures the strength of association between two variables; FDR: false discovery rate

Table S23: Correlation of lipoproteins with GlycA, GlycB, CRP, and Albumin intensity value.

	GlycA			GlycB			CRP			Albumin		
**Lipoproteins**	**rho**	**p-value**	**FDR**	**rho**	**p-value**	**FDR**	**rho**	**p-value**	**FDR**	**rho**	**p-value**	**FDR**
VLDL cholesterol (nmol/L)	0.18	0.224	0.937	-0.01	0.956	0.965	0.26	0.125	0.181	-0.08	0.684	0.684
IDL. cholesterol (nmol/L)	0.06	0.698	0.957	0.1	0.530	0.927	0.42	0.105	0.049	-0.57	<0.001	<0.001
LDL cholesterol (nmol/L)	-0.16	0.305	0.937	-0.08	0.582	0.927	0.2	0.233	0.275	-0.16	0.316	0.342
HDL cholesterol (nmol/L)	-0.17	0.278	0.937	-0.21	0.175	0.927	-0.44	0.007	0.049	0.66	<0.001	<0.001
VLDL triglyceride (nmol/L)	0.03	0.854	0.957	-0.12	0.431	0.927	0.28	0.938	0.163	-0.21	0.202	0.250
IDL triglyceride (nmol/L)	0.07	0.635	0.957	0.1	0.529	0.927	0.4	0.132	0.049	-0.57	<0.001	<0.001
LDL triglyceride (nmol/L)	0.01	0.996	0.966	0.06	0.713	0.927	0.38	0.189	0.062	-0.54	<0.001	<0.001
HDL triglyceride (nmol/L)	0.02	0.920	0.957	-0.01	0.956	0.965	0.11	0.522	0.565	-0.22	0.185	0.240
VLDL particle (nmol/L)	0.07	0.653	0.957	-0.07	0.630	0.927	0.3	0.762	0.142	-0.23	0.158	0.229
Large VLDL particle(nmol/L)	-0.02	0.872	0.957	-0.17	0.256	0.927	0.09	0.582	0.582	-0.18	0.285	0.322
Medium VLDL particle (nmol/L)	0.17	0.251	0.937	-0.08	0.618	0.927	0.17	0.319	0.361	0.12	0.452	0.470
Small VLDL particle(nmol/L)	0.06	0.711	0.957	-0.09	0.568	0.927	0.31	0.637	0.127	-0.25	0.129	0.197
LDL particle (nmol/L)	-0.16	0.279	0.937	-0.09	0.575	0.927	0.24	0.149	0.185	-0.29	0.760	0.124
Large LDL particle (nmol/L)	-0.03	0.856	0.957	-0.04	0.776	0.961	0.33	0.473	0.112	-0.18	0.268	0.317
Medium LDL particle (nmol/L)	-0.09	0.553	0.957	-0.02	0.901	0.965	0.27	0.104	0.169	-0.31	0.541	0.108
Small LDL particle (nmol/L)	-0.35	0.020	0.524	-0.26	0.904	0.927	0.1	0.568	0.582	-0.22	0.791	0.240
HDL particle (mol/L)	-0.16	0.283	0.937	-0.19	0.209	0.927	-0.42	0.944	0.049	0.67	<0.001	<0.001
Large HDL particle (mol/L)	0.09	0.547	0.957	-0.04	0.813	0.961	-0.26	0.121	0.181	0.49	<0.001	<0.001
Medium HDL particle (mol/L)	-0.05	0.760	0.957	-0.11	0.456	0.927	-0.31	0.625	0.127	0.37	<0.001	<0.001
Small HDL particle (mol/L)	-0.13	0.411	0.957	-0.17	0.267	0.927	-0.42	0.984	0.049	0.72	<0.001	<0.001
VLDL size (nm)	0.06	0.680	0.957	0.01	0.948	0.965	-0.25	0.133	0.181	0.4	<0.001	<0.001
LDL size (nm)	0.27	0.068	0.887	0.21	0.161	0.927	0.36	0.290	0.076	-0.29	0.713	0.124
HDL size (nm)	0.09	0.559	0.957	0.13	0.398	0.927	0.36	0.286	0.076	-0.69	<0.001	<0.001
Non-HDL particle (nmol/L)	-0.15	0.324	0.957	-0.08	0.587	0.927	0.25	0.143	0.185	-0.3	0.634	0.118
Total particle / HDL particle (nmol/L)	0.02	0.896	0.957	0.06	0.678	0.927	0.42	0.877	0.049	-0.59	<0.001	<0.001
LDL particle / HDL particle (nmol/L)	0.02	0.908	0.957	0.07	0.659	0.927	0.41	0.113	0.049	-0.58	<0.001	<0.001

Rho: spearman's correlation coefficient measures the strength of association between two variables; FDR: false discovery rate
